# Prospective associations of the infant gut microbiome and microbial function with social behaviors related to autism at age 3 years

**DOI:** 10.1038/s41598-020-72386-9

**Published:** 2020-09-23

**Authors:** Hannah E. Laue, Susan A. Korrick, Emily R. Baker, Margaret R. Karagas, Juliette C. Madan

**Affiliations:** 1 Department of Epidemiology, Geisel School of Medicine at Dartmouth College, Hanover, NH USA; 2 Department of Environmental Health, Harvard T.H. Chan School of Public Health, Boston, MA USA; 3 Channing Division of Network Medicine, Department of Medicine, Brigham and Women’s Hospital and Harvard Medical School, Boston, MA USA; 4 Department of Obstetrics and Gynecology, Dartmouth-Hitchcock Medical Center, Lebanon, NH USA; 5 Department of Pediatrics, Children’s Hospital at Dartmouth, Lebanon, NH USA; 6 Department of Psychiatry, Children’s Hospital at Dartmouth, Lebanon, NH USA

**Keywords:** Autism spectrum disorders, Epidemiology, Paediatric research, Autism spectrum disorders, Microbiota

## Abstract

The hypothesized link between gut bacteria and autism spectrum disorder (ASD) has been explored through animal models and human studies with microbiome assessment after ASD presentation. We aimed to prospectively characterize the association between the infant/toddler gut microbiome and ASD-related social behaviors at age 3 years. As part of an ongoing birth cohort gut bacterial diversity, structure, taxa, and function at 6 weeks (n = 166), 1 year (n = 158), 2 years (n = 129), and 3 years (n = 140) were quantified with 16S rRNA gene and shotgun metagenomic sequencing (n = 101 six weeks, n = 103 one year). ASD-related social behavior was assessed at age 3 years using Social Responsiveness Scale (SRS-2) T-scores. Covariate-adjusted linear and permutation-based models were implemented. Microbiome structure at 1 year was associated with SRS-2 total T-scores (*p* = 0.01). Several taxa at 1, 2, and 3 years were associated with SRS-2 performance, including many in the Lachnospiraceae family. Higher relative abundance of *Adlercreutzia equolifaciens* and *Ruminococcus torques* at 1 year related to poorer SRS-2 performance. Two functional pathways, l-ornithine and vitamin B6 biosynthesis, were associated with better social skills at 3 years. Our results support potential associations between early-childhood gut microbiome and social behaviors. Future mechanistic studies are warranted to pinpoint sensitive targets for intervention.

## Introduction

Autism spectrum disorder (ASD), a combination of disordered social behaviors and repetitive or restricted interests, affects one in 34 boys and one in 145 girls in the United States, with altered behaviors presenting in some children as early as 18 months of age or younger^1^, but most children are not diagnosed until after age three^2–8^. Early symptoms of ASD include poor eye contact, problems with social behaviors (difficulty with joint attention, no response to their name, poor nonverbal communication), and poor imitation skills; many children are not evaluated until families identify a delay in verbal communication^9^. Diagnosing ASD as early as infancy and resultant early intervention has been shown to improve neurodevelopmental outcomes, highlighting the importance of identifying potential biomarkers for early detection^10–12^. Even among neurotypical children a spectrum of behaviors exists^13^, some of which may create challenges in personal relationships and learning and professional environments. While several risk factors for ASD have been identified or hypothesized including genetics^14^, maternal infection during pregnancy^15–17^, and environmental exposures^18^, the etiologies are not well understood, leading to treatments targeting symptoms rather than underlying causes^19^. As a result, therapies, apart from early-life behavioral interventions, are often minimally effective^20,21^.

Recently, the interrelationship between the gastrointestinal tract and its microbiome (the compilation of microorganisms in the intestines) and the brain, known as the gut-brain axis, has been proposed as a potential cause or modifier of ASD behaviors^22–24^. Specifically, a recent mechanistic study found that mice transfected with the stool of ASD patients began displaying ASD behaviors, which were then modified by a correction of the imbalance in bacterial metabolites brought on by transfection^25^. Several case–control studies have compared the gut microbiome in children diagnosed with ASD to neurotypical siblings or community controls and observed notable differences^26–33^. However, these studies are subject to reverse causation, have been small, and have produced inconsistent results. To date, no epidemiological studies have examined the microbiome at multiple early-life ages to elucidate when in the lifespan it is most relevant to neurodevelopment, particularly autism-related social behaviors. Further, no prospective cohort study has employed metagenomic sequencing and examined bacterial function in relation to social behaviors to better understand how gut bacteria may affect the brain.

Our study aimed to address these gaps by examining the infant/toddler microbiome at four early-life time points, beginning at 6 weeks postpartum, in relation to a continuous measure of social behavior at 3 years of age. In addition to investigating bacterial diversity, structure, and specific taxa, we inferred gene function from metagenomic sequences to identify pathways associated with essential features of ASD.

## Results

### Population characteristics

Study populations were similar across the four time points; most mothers reported not smoking during pregnancy (> 95%) and approximately half were parous (Table 1). Most parents were not of advanced age (average maternal age at delivery 32.3 ± 4.6 years, average paternal age at delivery 33.6 ± 5.8 years). SRS-2 total T-scores were lower (better) and had a narrower distribution than the normative population (a nationally representative sample of 474 ratings of 247 children^34^) to which the test is standardized (i.e., approximate mean ± SD: 44 ± 5 in each study population compared to 50 ± 10 in the normative sample). As expected, within subject diversity increased as the microbiome sample age increased [mean ± SD Shannon Indices: 1.66 ± 0.49, 2.89 ± 0.56, 3.57 ± 0.46, and 3.83 ± 0.48 at 6 weeks, 1 year, 2 years, and 3 years, respectively; Table 1].

**Table 1:** Characteristics of New Hampshire Birth Cohort Study (NHBCS) subjects followed to age 3 years compared to those in each microbiome analytical cohort [N (%) or mean (± SD)].

	NHBCS subjects followed to age 3 years (n = 386)^a^	Six-week Cohort^b^ (n = 166)	One-year Cohort^b^ (n = 158)	Two-year Cohort^b^ (n = 129)	Three-year Cohort^b^ (n = 140)	Microbiome samples at all ages (n = 21)
**Parental characteristics**						
Parity						
Nulliparous	172 (44.6)	83 (50)^d^	77 (49)	64 (50)	61 (44)	12 (57)
Parous	214 (55.4)	83 (50)^d^	81 (51)	65 (50)	79 (56)	9 (43)
Maternal education						
College graduate or less	251 (65)	93 (56)	92 (58)	79 (61)	86 (61)	12 (57)
Any post-graduate education	135 (35)	73 (44)	66 (42)	50 (39)	54 (39)	9 (43)
Relationship status						
Married	355 (92)	156 (94)	150 (95)	120 (93)	132 (94)	19 (91)
Never married or separated	31 (8)	10 (6)	8 (5)	9 (7)	8 (6)	2 (9)
Maternal smoking during pregnancy						
No	372 (96.4)	160 (96)	154 (97)	126 (98)	135 (96)	20 (95)
Yes	14 (3.6)	6 (4)	4 (3)	3 (2)	5 (4)	1 (5)
Maternal age at delivery	32.3 (± 4.6)	32.3 (± 4.6)	32.4 (± 4.4)	32.6 (± 4.3)	32.7 (± 4.3)	32.6 (± 3.8)
Paternal age at delivery	33. 6 (± 5.8)	33.6 (± 5.9)	33.5 (± 5.7)	33.5 (± 5.4)	34.1 (± 5.7)	34.1 (± 3.9)
**Delivery characteristics**						
Delivery mode						
Vaginal	271 (70.2)	111 (67)	108 (68)	86 (67)	94 (67)	14 (67)
Caesarean	115 (29.8)	55 (33)	50 (32)	43 (33)	46 (33)	7 (33)
Peripartum antibiotic exposure						
Any	176 (45.6)	94 (57)^d^	81 (51)^d^	67 (52)	65 (46)	12 (57)
None	210 (54.4)	72 (43)^d^	77 (49)^d^	62 (48)	75 (54)	9 (43)
Gestational age (weeks)	39.5 (± 1.7)	39.4 (± 1.8)	39.5 (± 1.7)	39.5 (± 1.7)	39.5 (± 1.6)	39.9 (± 0.9)
Birth weight (g)	3,424 (± 539)	3,396 (± 546)	3,458 (± 569)	3,470 (± 572)	3,445 (± 503)	3,494 (± 391)
**Child characteristics**						
Feeding mode at 6 weeks						
Exclusively breast fed	206 (53.4)	90 (54)	84 (53)	71 (55)	79 (56)	10 (48)
Formula fed or mixed fed	180 (46.6)	76 (46)	74 (47)	58 (45)	61 (44)	11 (52)
Infant sex						
Male	199 (51.6)	86 (52)	90 (57)	79 (61)^d^	66 (47)	13 (62)
Female	187 (48.4)	80 (48)	68 (43)	50 (39)^d^	74 (53)	8 (38)
Age at SRS-2^d^ follow-up (years)	3.1 (± 0.2)	3.1 (± 0.3)	3.1 (± 0.3)	3.1 (± 0.3)	3.1 (± 0.2)	3.1 (± 0.3)
SRS-2^d^ total T-Score (unitless)	43.7 (± 4.7)	43.5 (± 4.6)	43.2 (± 4.5)	43.6 (± 4.4)	43.9 (± 4.5)	43.3 (± 4.3)
Microbial diversity						
Shannon Index (unitless)	–	1.66 (± 0.49)	2.89 (± 0.56)	3.57 (± 0.46)	3.83 (± 0.48)	–
Simpson Index (unitless)	–	0.68 (± 0.16)	0.88 (± 0.09)	0.94 (± 0.04)	0.95 (± 0.03)	–
Taxa count (#)	–	33.1 (± 11.8)	90.1 (± 35.1)	166.2 (± 45.1)	208.2 (± 67.6)	–

^a^Subjects followed to 3 years with complete covariate information (273 have microbiome sequenced in at least one stool sample).

^b^Overlap occurs between cohorts.

^c^Statistically different from complete cases not included in the analysis (*p* < 0.05).

^d^Social Responsiveness Scale, 2nd edition.

### Within-subject diversity

**Figure 1:**
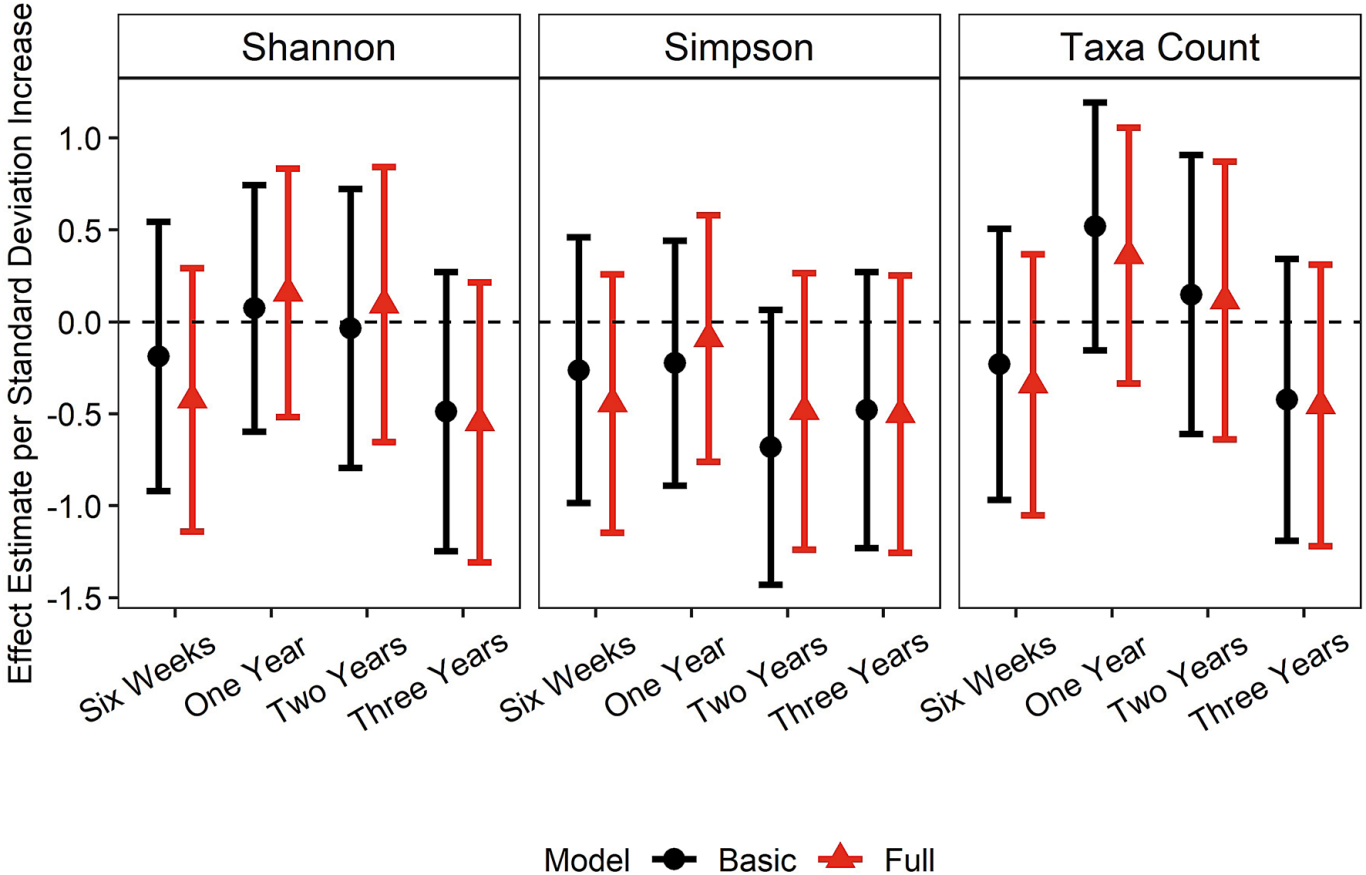
Associations of within-subject diversity with total T-scores on the Social Responsiveness Scale-2 (SRS-2) at 3 years of age. Basic models adjust for age at SRS-2, maternal education, marital status, maternal age, paternal age, child sex. Full models adjust for all covariates in the basic model and maternal self-reported smoking during pregnancy, early exclusive breastfeeding, delivery mode, peripartum antibiotics, and gestational age.

At most time points, increased within-subject diversity was correlated with improved SRS-2 total T-scores [β_Six-week Shannon_ = − 0.19 (− 0.92, 0.54), *p* = 0.62; β_One-year Shannon_ = 0.07 (− 0.60, 0.74), *p* = 0.83; β_Two-year Shannon_ = − 0.04 (− 0.79, 0.72), *p* = 0.93; β_Three-year Shannon_ =  − 0.49 (− 1.25, 0.27), *p* = 0.21 in basic models], although the relationships were not statistically significant (Fig. 1, Supplementary Table S1). Estimates were similar between basic and full models, but within-subject diversity at 6 weeks appeared to be more strongly correlated with SRS-2 total T-scores in the full model. Sensitivity analyses did not alter the conclusions (Supplementary Tables S2–S7). Using metagenomics data, all metrics of within-subject diversity at 1 year related to improved social behaviors. However, at 6 weeks higher Shannon and Simpson Indices were associated with better social behaviors, but higher taxa count related with worse social behavior (Supplementary Table S8). None of the estimates for metagenomic within-subject diversity reached statistical significance.

### Microbial community structure

**Figure 2:**
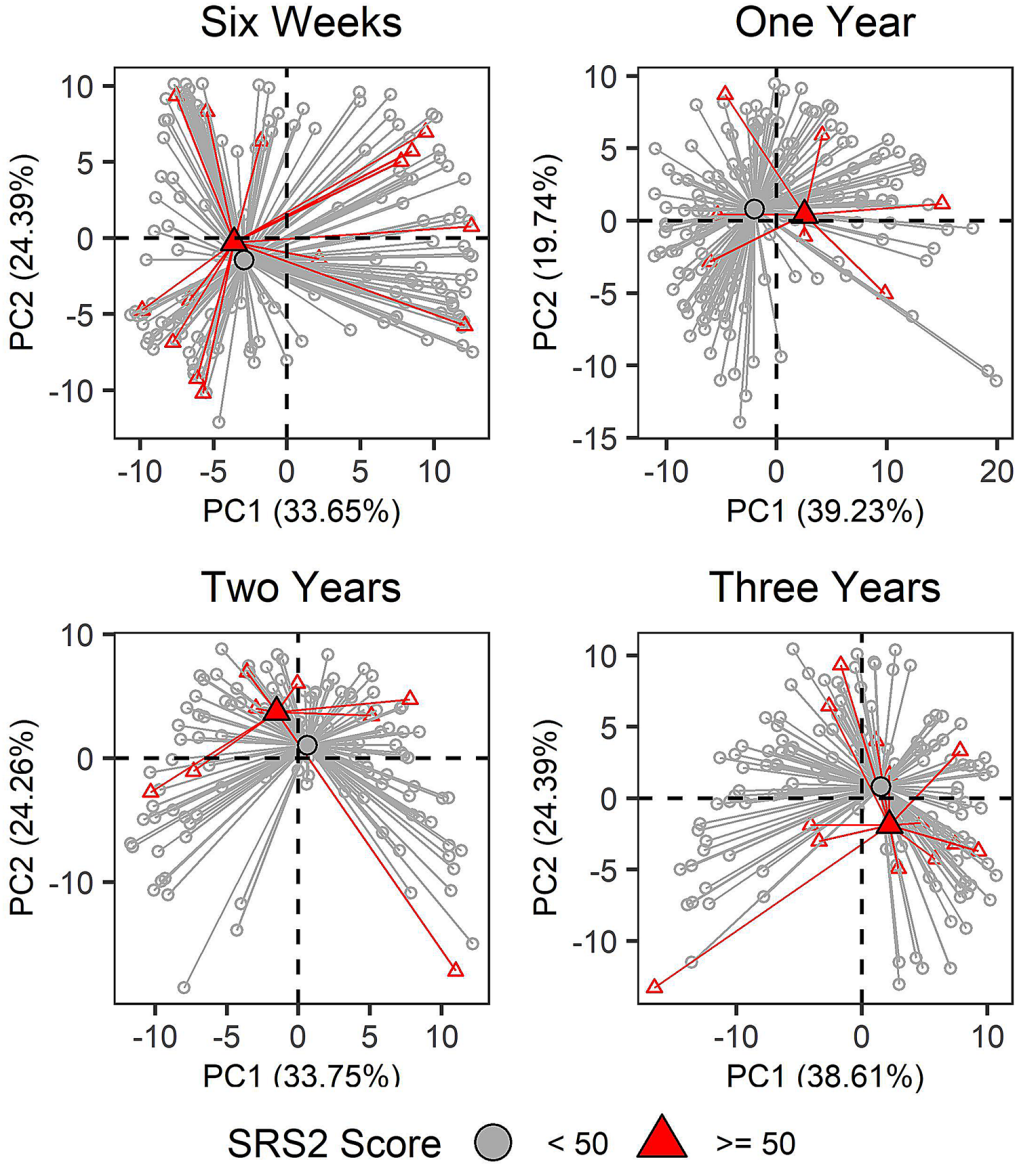
Generalized Unifrac distance principal component (PC) plots. Each point represents a subject and lines indicate distance to median centroids for subjects with Social Responsiveness Scale-2 (SRS-2) total T-scores above and below 50. Some points were removed for visual clarity (4 at one year and 1 at three years) but contributed to centroid calculation. Associations with continuous SRS-2 scores were statistically significant at 1 year (*p* = 0.01, 0.06 in basic and full Models, respectively). Principal components are time point specific. The contribution of each principal component to overall variability in community structure is found along the axis (%).

Community structure of the gut microbiome at 1 year was associated with total SRS-2T-scores at 3 years, particularly in the basic model (*p* = 0.01, 0.06 in basic and full models, respectively; Fig. 2, Supplementary Table S9). The *p* values were similar between the basic and full models, except for 2-year community structure, where birth mode and peripartum antibiotic exposure explained significant portions of the variability in the full model (data not shown). Similar results were obtained in sensitivity analyses (Supplementary Table S10).

### Individual taxa

The relative abundance of specific taxa at 1 year, 2 years, and 3 years were associated with social behaviors (Table 2). Higher relative abundance of two ASVs in the Lachnospiraceae (*Blautia producta* and an unknown taxon) at 1 year were associated with worse social behavior at 3 years [e.g., β_*B.producta-*full_ = 0.29 (0.15, 0.42), q = 0.017]. Similarly, higher relative abundances of *Coprococcus* and *Bifidobacterium* at 2 years were associated with poorer social behaviors in both basic and full models. Two additional taxa (*Ruminococcus gnavus* and *Sutterella*) were associated with SRS-2 total T-scores in only the basic and full models, respectively [e.g., β_*R.gnavus-*basic_ = 0.17 (0.08, 0.26), q = 0.069]. At 3 years, higher relative abundance of *Butyricicoccus pullicaecorum* was associated with worse social behavior in both basic and full models. In general, associations were modest, and the relative abundances of associated taxa were low (Table 2). Most of the statistically significantly associated taxa were in the Lachnospiraceae family (e.g., *Blautia producta*, *Coprococcus*), or more broadly in the Clostridiales order in the Firmicutes phylum (e.g., *Butyricicoccus pullicaecorum*), which was also true of the taxa with a nominal *p *value < 0.05 (Supplementary Table S11). Sensitivity and supplemental analysis results were similar (Supplementary Tables S12,S13).

**Table 2:** Taxa associated with Social Responsiveness Scale-2 (SRS-2) total T-scores at 3 years (q < 0.25).

Sequencing method	Microbiome age	Family	Genus	Species	Estimate (q) in basic model^a^	Estimate (q) in full model^a^	Average relative abundance (%)^b^
16S rRNA	Six weeks	NA					
	One year	Lachnospiraceae	Blautia	producta	0.34 (0.01)	0.29 (0.017)	0.1
		Lachnospiraceae			0.01 (0.201)	–^c^	0.01
	Two years	Lachnospiraceae	Coprococcus		0.07 (0.03)	0.06 (0.146)	0.05
		Lachnospiraceae	[Ruminococcus]	gnavus	0.17 (0.069)	–^c^	0.2
		Bifidobacteriaceae	Bifidobacterium		0.06 (0.03)	0.05 (0.148)	0.03
		Alcaligenaceae	Sutterella		–^c^	0.01 (0.211)	0.01
	Three years	Ruminococcaceae	Butyricicoccus	pullicaecorum	0.01 (0.072)	0.01 (0.066)	0.02
Metagenomics	Six weeks	Ruminococcaceae	Flavonifactor	plautii	0.002 (0.052)	–^c^	0.11
	One year	Coriobacteriaceae	Adlercreutzia	equolifaciens	0.002 (0.004)	0.002 (0.001)	0.07
		Lachnospiraceae	[Ruminococcus]	torques	0.01 (0.01)	0.01 (0.042)	1.13
		Lachnospiraceae		bacterium_6_1_63FAA	0.001 (0.01)	0.001 (0.067)	0.04
		Erysipelotrichaceae	Eubacterium	dolichum	0.0003 (0.038)	–^c^	0.02

^a^MaAsLin models the microbiome as the outcome. Thus, estimate is increase in % relative abundance per point increase on SRS-2 total T-score.

^b^Mean relative abundance of specific amplicon sequence variant given microbiome age.

^c^Empty estimate cell indicates that the estimate was not significant (q < 0.25) in the given model.

Metagenomic data provided deeper insight into the taxa whose relative abundance at 6 weeks and 1 year were associated with SRS-2 total T-scores (Table 2). At 6 weeks, a taxon identified as *Flavonifactor plautii* was associated with worse total T-scores on the SRS-2. Similarly, at 1 year greater relative abundance of four taxa were associated with poorer social behavior, namely *Adlercreutzia equolfaciens*, *Ruminococcus torques*, *Eubacterium dolichum*, and a bacterium in the Lachnospiraceae, [e.g., β_*A.equolifaciens-*full_ = 0.002 (0.001, 0.002), q = 0.001]. Associations between taxa and SRS-2 total T-scores reaching a nominal *p* value of 0.05 were largely in the Actinobacteria (class) and Clostridiales (Supplementary Table S14).

### Bacterial functional pathways

**Figure 3:**
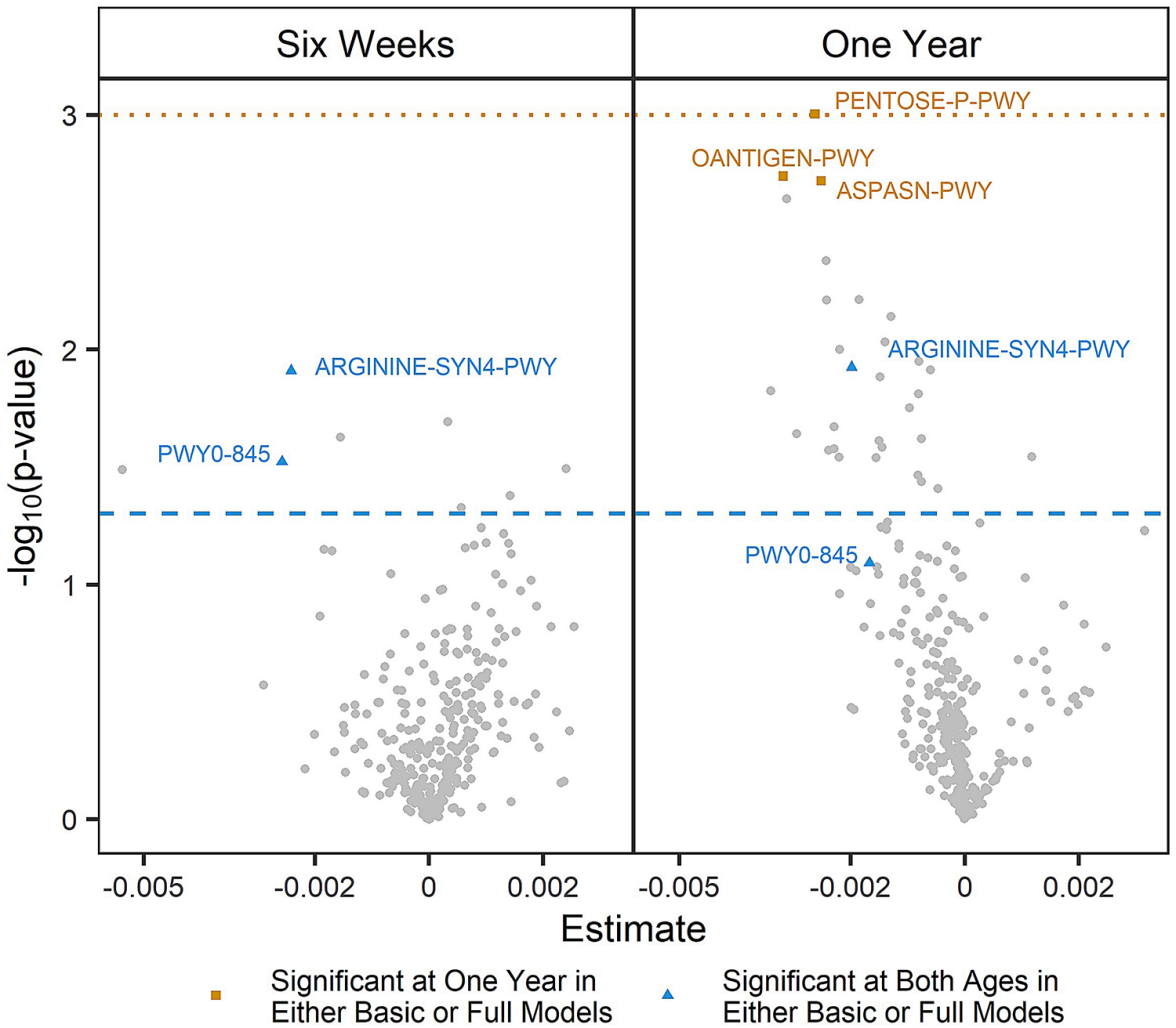
Associations of bacterial functional pathways and Social Responsiveness Scale-2 (SRS-2) total T-scores. Volcano plots of associations between metagenomically identified functional pathways and SRS-2 total T-scores in full models (adjusting for age at SRS-2, maternal education, marital status, maternal age, paternal age, child sex, maternal self-reported smoking during pregnancy, early exclusive breastfeeding, delivery mode, peripartum antibiotics, and gestational age). Points representing unmapped or unintegrated pathways have been removed for visual clarity (2 points per plot). Dashed blue line indicates *p* = 0.05, dotted orange line indicates *p* = 0.001. ARGININE-SYN4-PWY: l-ornithine de novo biosynthesis; ASPASN-PWY: superpathway of l-aspartate and l-asparagine synthesis; OANTINGEN-PWY: O-antigen building blocks biosynthesis (*Escherichia coli*); PENTOSE-P-PWY: pentose phosphate pathway; PWY0-845: superpathway of pyridoxal 5′-phosphate biosynthesis and salvage.

Increased relative abundance of two functional pathways, l-ornithine de novo biosynthesis and the superpathway of pyridoxal 5′-phosphate biosynthesis and salvage, at both 6 weeks and 1 year were associated with better social behavior at 3 years [e.g., β_l-ornithine-6W*-*basic_ = − 2.4E−5 (− 4.2E−5, − 6.8E−6), *p* = 0.008; Fig. 3, Supplementary Figure S1, Supplementary Table S15]. An additional three pathways (the superpathway of l-aspartate and l-asparagine synthesis, O-antigen building blocks biosynthesis—*Escherichia coli*, and pentose phosphate pathway) had highly significant associations (*p* < 0.001) at 1 year in either basic or full models (Fig. 3, Supplementary Figure S1). Several associated pathways (*p* < 0.05) are involved in the urea cycle (involving aspartate, asparagine, or ornithine), pyridoxal 5′-phosphate (highly bioavailable vitamin B6) synthesis, or menaquinol biosynthesis (Supplementary Table S15).

## Discussion

Our analysis uncovered the potential for several novel, time-dependent associations between the early-life gut microbiome and child social behaviors at age three. Specifically, we found suggestive evidence of better social behaviors with increased within-subject microbial diversity during infancy and early childhood as well as an association with bacterial community structure at 1 year of life. Many of the taxa associated with relative decrements in social behaviors were in the Lachnospiraceae family. Metagenomic sequencing data from 6-week and 1-year fecal samples complemented our 16S rRNA findings and demonstrated several associations, including with *Adlercreutzia equolifaciens* and *Ruminococcus torques*. By classifying our metagenomic sequences into functional pathways, we also identified several promising pathways through which the microbiome may act on autism-related social behaviors.

Studies of within-subject diversity and health outcomes including attention deficit hyperactivity disorder^35^ and gastrointestinal disorders^36^, both common ASD comorbidities^37,38^, generally have found a beneficial association with increased microbial diversity. Our limited evidence of an association between within-subject diversity and social behaviors supports the potential for the microbiome to interact with the brain and behavior at a different level (e.g., the taxon level). The direction of the effect estimates in our study was somewhat sensitive to the metric used, with the Simpson Index (a measure of evenness) providing consistent evidence of potential beneficial impacts across time points and the Shannon Index (a measure of richness and evenness) and taxa count (a measure of richness) indicating a potential adverse association between increased diversity and social behaviors at 1 and 2 years^39^. However, the lack of statistical significance implies our findings should be interpreted carefully.

The association with bacterial community structure was strongest when measured at 1 year, when the bacterial community is more established (as compared to 6 weeks) but more malleable than an adult microbiome^40^. However, because not all subjects appear in each analysis any time-specific effects may be artifacts of the specific population. Similar to the within-subject diversity analysis, the association between bacterial community structure and social behaviors did not appear to differ with the addition of autism risk factors for which community structure may act as a mediator.

Several taxa at 1, 2, and 3 years were associated with poorer social behaviors, primarily in the Lachnospiraceae family, one of the most abundant in the gut. While the family was altered in several case–control investigations of ASD, the direction is not consistent across studies^27–29^, and is not present in others^30,31^. A prospective study examining the gut microbiome at six months and scores on the Ages and Stages Questionnaire (ASQ) at 3 years in the Vitamin D Antenatal Asthma Reduction Trial (n = 309 primarily neurotypical children) found an adverse association between *Ruminococcus*, a genus in the Lachnospiraceae that was also identified in our analyses, and both total ASQ score and personal/social development^41^. We observed an association between a low-abundance *Bifidobacterium* taxon at two years and worse social behaviors. *Bifidobacterium* species are generally considered to be beneficial with respect to their psychoactive properties, especially in infants and children^42–45^. Because this finding was not consistent across time points or in our more probing shotgun metagenomics analyses it may be the result of chance. In our analysis using metagenomic data, which allows more precise identification of bacterial species and strain, we identified an adverse association between *Ruminococcus torques*, previously found to be increased in ASD cases with gastrointestinal symptoms (n = 54), and SRS-2 performance^46^. We also found an association between *Adlercreutzia equolifaciens* and poorer social behaviors. Although this taxon has not been detected in other ASD studies, its primary product, a phytoestrogen called equol, may interfere with normal microglial function^47^, including essential early-life synaptic pruning, a deficit of which is a putative mechanism for ASD^48,49^. Indeed, one recent study found increased genistein and daidzein (two other phytoestrogens dependent on gut microbial production) in mice colonized with ASD-patient microbiomes compared to controls^25^. Importantly, ASD phenotypes, including altered social behaviors, were reduced after restoring gut microbiome metabolomic balance.

In addition to more precisely identifying associations with select taxa, the use of metagenomic data allowed us to infer functional pathways the enrichment or depletion of which was associated with worse social behaviors. Two pathways were less abundant in children with better social behaviors in nearly all models: the l-ornithine de novo biosynthesis pathway and the superpathway of pyridoxal 5′-phosphate biosynthesis and salvage. In humans, ornithine is essential for the detoxification of ammonia, a neurotoxin, to urea^50–52^. Our findings are supported by a case–control study of ASD that found decreased urinary ornithine among cases provided that less excretion is an indication of increased salvage^53^. Although the authors of this study aspired to identify predictive biomarkers of ASD, their biospecimens, collected at an average of 5 years, were subject to reverse causation (i.e., autistic behaviors, with onset by 18 months^3–5^, may impact diet or exposures, thus altering urinary amino acid concentrations). In contrast, our study indicates that as early as 6 weeks of life alterations in gut bacterial ornithine production are associated with social behaviors in childhood. The relative abundance of another amino acid synthesis pathway (superpathway of l-aspartate and l-asparagine synthesis) at 1 year was also associated with SRS-2 performance. Asparagine is essential to brain development and function^54^. Thus, further investigation of this pathway in ASD may be informative.

Our findings regarding the superpathway of pyridoxal 5′-phosphate (highly available vitamin B6) biosynthesis and salvage are relevant to clinical trials examining the effectiveness of high-dose vitamin B6 and magnesium supplementation in reducing autistic symptoms. While most of these studies have been small, and with conflicting results^55–59^, a more recent study suggests that a certain phenotypic subset of autistic subjects respond to B6 and magnesium supplementation^60^. Further research is needed to clarify whether/what gut microbial functions can identify responders so that treatment can be more successfully targeted. At 1 year we also found associations with O-antigen building blocks biosynthesis and pentose phosphate pathway. O-antigens are a vital component of lipopolysaccharides, which are hypothesized contributors to ASD, and demonstrated effectors of autism phenotypes^61–63^. The implications of the observed association with the pentose phosphate pathway, which is essential to glucose metabolism, are less clear. By generating nicotinamide adenine dinucleotide phosphate (NADPH) this pathway may contribute to the detoxification of reactive oxygen species, but this hypothesis must be robustly tested with in vitro models before any conclusion can be reached^64^.

Although this study suggests potential windows of microbial development that are sensitive for the development of social behaviors, our findings could have been population-specific rather than true effects because the populations comprise different individuals at each time point. In addition, despite our adjustment for numerous important confounders, we were not able to account for others including genetics. We chose not to include later antibiotic exposure in our models due to its lack of association with SRS-2 scores. However, this may have resulted in nondifferential exposure misclassification, adding imprecision to our estimates, but not biasing them. Further, due to limitations of statistical methods available for microbiome analysis (*e.g.*, treating the microbiome as the dependent variable when it is the hypothesized exposure) and the constraints of observational data our findings cannot be interpreted as causal. However, as the first analysis to prospectively examine the microbiome at multiple time points early in life in relation to social development we have identified several important potential associations that should be explored further in mechanistic models.

The strengths of our study include careful adjustment for a range of variables that may confound the association between the microbiome and SRS-2 total T-scores or predict autism-like behavior. Further, by considering a model including variables for which the microbiome may act as a mediator we found that the association of the microbiome with social behaviors was largely independent of potential risk factors for autism (*i.e.*, variables in the full model). Other variables—such as dietary factors and probiotics—were considered, but ultimately rejected, for model inclusion because of the likelihood of reverse causation (i.e., autism-related behaviors are known to affect food selectivity)^65^. Ours is among one of the largest studies to date to consider the microbiome and autism-related behaviors, particularly in a prospective cohort setting . Our finding of differences as early as 6 weeks of life highlights opportunities for interventions that have been shown to change the direction of neurodevelopment in murine models^25^. Additionally, our findings in a pregnancy cohort are generalizable to non-clinical populations while still informing autism research. Specifically, because our cohort comprises primarily neurotypical children we expect stronger associations in a more neurodiverse population.

In summary, our findings suggest an association of the infant/toddler gut microbiome with social behaviors in a US population sample. Further research is warranted to elucidate mechanisms, and to determine whether features of the gut microbiome can be used to identify ASD during a vulnerable window during which time interventions can optimize neurodevelopmental outcomes.

## Methods and materials

### Study cohort

The New Hampshire Birth Cohort Study, an ongoing prospective pregnancy cohort, recruited pregnant women (ages 18–45) as previously described (Supplementary Methods)^66^. This analysis is based on a subset of children who provided a stool sample at 6 weeks, 1 year, 2 years, or 3 years and completed a social behavioral assessment at 3 years (n = 273, Supplementary Figure S2, Supplementary Table S16). Although subjects overlapped in each of the populations, few (n = 21) had microbiome sequencing data at all four time points. Parents of participants provided written informed consent. Study protocols were reviewed and approved by the Center for the Protection of Human Subjects at Dartmouth, and all methods were carried out in accordance with relevant guidelines and regulations.

### Stool sample collection and microbial sequencing

Stools were collected from infants/toddlers at 6 weeks, 1 year, 2 years, and 3 years postpartum. If the child was using diapers, the sample was collected by parents from a study-provided diaper, as previously described^67^. If the child was no longer using diapers, parents were provided an acid-washed receptacle that fit into the toilet to collect a urine-free stool sample (Supplementary Methods). Established protocols were followed for 16S rRNA gene sequencing and data processing (Supplementary Methods)^67–72^. A subset of DNA samples (from 6-week and 1-year stools) also underwent metagenomic sequencing at Marine Biological Laboratory as previously described (Supplementary Methods)^73–75^.

### Assessment of social behaviors

Approximately 3 years postpartum, parents were asked to complete the Social Responsiveness Scale, 2nd edition, preschool form (SRS-2), which asks about their child’s usual behavior^34,76^. The SRS-2 is a standardized, validated instrument designed to assess social behavioral deficits and autistic traits as reflected in five components of social behavior (awareness, cognition, communication, motivation, and restricted interests/repetitive behavior) on a continuous scale^77^. SRS-2 provides raw and age-standardized T-scores for each subscale as well as an overall total score. The T-scores are standardized to a mean (SD) of 50 (10) with higher scores indicating poorer social behavioral skills. For some visual representations of results scores were dichotomized at 50 points (approximately 1 SD above our population mean, 5–10% of each study population above), but continuous total T-scores were used for all primary analyses. In clinical settings a more stringent cut off of 60 points is applied to identify individuals with mild social impairment.

### Statistical analysis

Preliminary analyses identified factors associated with SRS-2 total T-scores in the study population and covariates were then selected if they were associated with SRS-2 scores (and in some cases the microbiome), but unlikely to be a factor for which the microbiome acts as a mediator. These included child age at follow-up, maternal education, maternal marital status, parity, maternal and paternal age at delivery, and child sex—the basic model. Additional models were run including autism risk factors for which the microbiome may act as a partial mediator (basic model variables and maternal smoking during pregnancy^78^, early postnatal exclusive breastfeeding^79–81^, delivery mode^82^, perinatal antibiotic exposure^83^, and continuous gestational age at delivery^84^—the full model).

Shannon and Simpson Indices and taxa count were linearly regressed against SRS-2 total T-scores adjusting for covariates included in the basic and full models. The Shannon Index is reported as the primary within-subject diversity metric because it accounts for both richness and evenness of species^85^. Bacterial community structure was contrasted between subjects with generalized UniFrac (GUniFrac) distances^86^. To assess the significance of the relation between bacterial community structure and SRS-2 total T-scores we employed the *adonis2* function in the “vegan” package with 10,000 permutations^87^. Multivariate Association with Linear Models (MaAsLin2) was used to determine the association between individual amplicon sequence variants (ASVs) and total SRS-2T-scores^88,89^. We restricted our analyses to ASVs with at least 0.001% relative abundance in at least 10% of subjects to reduce multiple testing. Any taxon-level association with an FDR-corrected q-value < 0.25 was considered statistically significant, which is the MaAsLin default and is commonly used in microbiome studies where further investigation and validation of results is necessary^90,91^. A complementary analysis using diversities and taxon relative abundances derived from metagenomic data was conducted at 6 weeks and 1 year. Pathway relative abundances associated with SRS-2 total T-scores were determined with MaAsLin2, restricting to pathways with at least 0.0001% in at least 10% of samples. Due to the exploratory nature of this analysis, all pathways with a nominal *p* value < 0.05 were deemed of interest. Sensitivity analyses are described in Supplementary Methods.

## Supplementary information

Supplementary information.

## Data Availability

The 16S rRNA gene sequencing and shotgun metagenomic data used in this study are available through the National Center for Biotechnology Information (NCBI) Sequence Read Archive: https://ncbi.nlm.nih.gov/sra under accession number PRJNA296814.
