# Supplementary material for: Prospective associations of the infant gut microbiome and microbial function with social behaviors related to autism at age 3 years
Source: Sci Rep. 2020 Sep 23;10:15515. doi: 10.1038/s41598-020-72386-9 (PMC7511970; doi:10.1038/s41598-020-72386-9)

Prospective associations of the infant gut microbiome and microbial function with social behaviors related to autism at age three years

Hannah E. Laue[1*], Susan A. Korrick[2,3], Emily R. Baker[4], Margaret R. Karagas[1], Juliette C. Madan[1,5]

[1] Department of Epidemiology, Geisel School of Medicine at Dartmouth, Hanover, NH, USA; Hannah.E.Laue@Dartmouth.edu, Margaret.R.Karagas@Dartmouth.edu; Juliette.C.Madan@Hitchcock.org

[2] Department of Environmental Health, Harvard T.H. Chan School of Public Health, Boston, MA, USA; Susan.Korrick@Channing.Harvard.edu

[3] Channing Division of Network Medicine, Department of Medicine, Brigham and Women's Hospital and Harvard Medical School, Boston, MA, USA

[4] Department of Obstetrics and Gynecology, Dartmouth-Hitchcock Medical Center, Lebanon, NH, USA; Emily.R.Baker@Hitchcock.org

[5] Departments of Pediatrics and Psychiatry, Children's Hospital at Dartmouth, Lebanon, NH, USA

Supplementary Methods

New Hampshire Birth Cohort Study details

Recruitment occurred at New Hampshire prenatal clinics at approximately 24-28 weeks gestation. Women were included if they used a private, unregulated well at their residence since their last menstrual period and had no plans to move.

Stool sample processing

Stool samples were stored in participants' home freezer until they were able to return it to the study site (within 24 hours). Stool was then thawed at 4°C so that it could be aliquoted into cryovial tubes containing RNAlater and homogenized before long-term storage at -80°C. DNA was extracted from these stools and both 16S rRNA and metagenomic sequencing were performed.

16S rRNA gene sequencing

Sequencing was performed on an Illumina MiSeq at the Marine Biological Laboratory (MBL) in Woods Hole, Massachusetts [1–3]. Sequences were filtered and trimmed to amplicon sequence variants (ASVs) using the DADA2 pipeline and paired to taxonomies inferred from the Greengenes database [1,4–6]. Due to preliminary ASV-specific results and the hypothesized biological importance of the relative abundance of Bacteroidetes compared to the relative abundance of Firmicutes, this ratio was calculated and used as an outcome in a supplemental analysis [7,8]. Within-subject diversity was assessed using the Shannon [9] and Simpson [10] Indices as well as the count of detected ASVs.

Metagenomic sequencing

Briefly, DNA was sheared to a mean length of 400bp before construction of sequencing libraries following Nugen's Ovation Ultralow V2 protocol and sequencing on an Illumina NextSeq. After

trimming and cleaning reads, taxa were identified using MetaPhlAn [11] and pathway relative abundance was inferred using the HUMAnN2 pipeline with default parameters [12].

Sensitivity analyses

Primary analyses used SRS-2 total T-scores at three years, but associations with raw scores were tested in sensitivity analyses. In other sensitivity analyses, in a subset of participants with available data, we considered third trimester urinary cotinine (comparing detect to non-detect) in place of maternal self-report of smoking. We also conducted an analysis restricted to term infants. Additional sensitivity analyses considered the inclusion of covariates such as an indicator variable for any breastfeeding at six months and a measure of the parent-child relationship (Parenting Relationship Questionnaire-preschool form (PRQ), see below) [13]. The PRQ was included in basic and full models at all time points, whereas six-month breastfeeding was included in basic and full models for the six-week microbiome, but only in full models for the later microbiome ages because it may be a factor for which subsequent microbiome composition acts as a mediator. To explore the possibility that any observed time-specific associations were the result of population-specific effects (*i.e.*, due to selection bias), an analysis was conducted among children with microbiome sequencing available at all time points (n = 21; **Supplementary Table S1**).

Incorporation of the Parenting Relationship Questionnaire (PRQ)

The preschool version of the PRQ produces five scores assessing different domains of parenting quality including attachment, discipline practices, involvement, parenting confidence, and relational frustration. Each raw score is standardized to a reference population producing T-scores with an average of 50 and a standard deviation of 10. For four of these scales (attachment, discipline practices, involvement, and parenting confidence) a higher score is considered better whereas a higher relational frustration score is considered worse. To

incorporate these five scales as one covariate the relational frustration score was transformed as follows:

$$Relational\ Frustration_{Transformed} = 100 - Relational\ Frustration \qquad \text{(Eq. 1)}$$

The five scores (attachment, discipline, practices, involvement, parenting confidence, and transformed relational frustration) were summed after confirming the scores had similar distributions in our population.

References

1. Madan, J. C. *et al.* Association of Cesarean Delivery and Formula Supplementation With the Intestinal Microbiome of 6-Week-Old Infants. *JAMA Pediatr.* **170**, 212–219 (2016).
2. Caporaso, J. G. *et al.* Ultra-high-throughput microbial community analysis on the Illumina HiSeq and MiSeq platforms. *ISME J.* **6**, 1621–1624 (2012).
3. Degnan, P. H. & Ochman, H. Illumina-based analysis of microbial community diversity. *ISME J.* **6**, 183–194 (2012).
4. Hoen, A. G. *et al.* Sex-specific associations of infants' gut microbiome with arsenic exposure in a US population. *Sci. Rep.* **8**, 12627 (2018).
5. Callahan, B. J. *et al.* DADA2: High-resolution sample inference from Illumina amplicon data. *Nat. Methods* **13**, 581–583 (2016).
6. DeSantis, T. Z. *et al.* Greengenes, a chimera-checked 16S rRNA gene database and workbench compatible with ARB. *Appl. Environ. Microbiol.* **72**, 5069–5072 (2006).
7. Ley, R. E., Turnbaugh, P. J., Klein, S. & Gordon, J. I. Microbial ecology: human gut microbes associated with obesity. *Nature* **444**, 1022–1023 (2006).
8. Mariat, D. *et al.* The Firmicutes/Bacteroidetes ratio of the human microbiota changes with age. *BMC Microbiol.* **9**, 123 (2009).
9. Shannon, C. & Weaver, W. *The Mathematical Theory of Communication*. (The University of Illinois Press).
10. Simpson, E. H. Measurement of Diversity. *Nature* **163**, 688 (1949).
11. Segata, N. *et al.* Metagenomic microbial community profiling using unique clade-specific marker genes. *Nat. Methods* **9**, 811–814 (2012).
12. Franzosa, E. A. *et al.* Species-level functional profiling of metagenomes and metatranscriptomes. *Nat. Methods* **15**, 962–968 (2018).
13. Rubinic, D. & Schwickrath, H. Test Review: Parenting Relationship Questionnaire. *J. Psychoeduc. Assess.* **28**, 270–275 (2010).

Supplementary Table S1. Difference in Social Responsiveness Scale-2 total T-score per standard deviation[a] increase in within-subject diversity

Microbiome Age	Shannon				Simpson				Taxa Count			
	Basic Model[b]		Full Model[c]		Basic Model		Full Model		Basic Model		Full Model	
	Estimate (95% CI)	p	Estimate (95% CI)	p	Estimate (95% CI)	p	Estimate (95% CI)	p	Estimate (95% CI)	p	Estimate (95% CI)	p
Six Weeks	-0.19 (-0.92, 0.54)	0.62	-0.42 (-1.14, 0.29)	0.25	-0.26 (-0.99, 0.46)	0.48	-0.44 (-1.15, 0.26)	0.22	-0.23 (-0.97, 0.51)	0.54	-0.34 (-1.05, 0.37)	0.35
One Year	0.07 (-0.60, 0.74)	0.83	0.11 (-0.57, 0.79)	0.75	-0.22 (-0.89, 0.44)	0.51	-0.14 (-0. 82, 0.54)	0.68	0.52 (-0.15, 1.19)	0.13	0.36 (-0.33, 1.06)	0.31
Two Years	-0.04 (-0.79, 0.72)	0.93	0.09 (-0.65, 0.84)	0.80	-0.68 (-1.43, 0.07)	0.08*	-0.49 (-1.24, 0.27)	0.21	0.15 (-0.61, 0.91)	0.70	0.12 (-0.64, 0.87)	0.76
Three Years	-0.49 (-1.25, 0.27)	0.21	-0.55 (-1.30, 0.21)	0.16	-0.48 (-1.23, 0.27)	0.21	-0.50 (-1.26, 0.25)	0.19	-0.42 (-1.19, 0.34)	0.28	-0.45 (-1.22, 0.31)	0.24

[a] Standard deviations for Shannon Index: 0.49, 0.56, 0.46, 0.48 for six-week, one-year, two-year, and three-year microbiomes respectively. Standard deviations for Simpson Index: 0.16, 0.09, 0.04, 0.03 for six-week, one-year, two-year, and three-year microbiomes respectively. Standard deviations for Taxa Count: 11.8, 35.1, 45.1, 67.6 for six-week, one-year, two-year, and three-year microbiomes respectively.

[b] Basic model adjusts for age at SRS-2, maternal education, marital status, maternal age, paternal age, and child sex

[c] Full model adjusts for basic model + maternal self-reported smoking during pregnancy, early exclusive breastfeeding, delivery mode, peripartum antibiotics, and gestational age

* indicates $p < 0.1$

Supplementary Table S2. Difference in Social Responsiveness Scale-2 total raw score per standard deviation[a] increase in within-subject diversity

Microbiome Age	Shannon				Simpson				Taxa Count			
	Basic Model[b]		Full Model[c]		Basic Model		Full Model		Basic Model		Full Model	
	Estimate (95% CI)	p	Estimate (95% CI)	p	Estimate (95% CI)	p	Estimate (95% CI)	p	Estimate (95% CI)	p	Estimate (95% CI)	p
Six Weeks	-0.43 (-2.31, 1.46)	0.66	-1.01 (-2.86, 0.83)	0.28	-0.63 (-2.50, 1.23)	0.51	-1.08 (-2.89, 0.73)	0.24	-0.54 (-2.43, 1.35)	0.58	-0.94 (-2.75, 0.88)	0.31
One Year	0.19 (-1.54, 1.93)	0.83	0.27 (-1.50, 2.04)	0.77	-0.55 (-2.28, 1.17)	0.35	-0.35 (-2.10, 1.40)	0.70	1.38 (-0.37, 3.12)	0.12	0.94 (-0.85, 2.76)	0.30
Two Years	-0.16 (-2.13, 1.81)	0.87	0.18 (-1.77, 2.13)	0.86	-1.87 (-3.81, 0.07)	0.06*	-1.36 (-3.31, 0.60)	0.18	0.32 (-1.65, 2.30)	0.75	0.24 (-1.72, 2.21)	0.81
Three Years	-1.15 (-3.11, 0.82)	0.25	-1.30 (-3.26, 0.67)	0.20	-1.18 (-3.11, 0.76)	0.24	-1.23 (-3.18, 0.72)	0.22	-0.99 (-2.97, 0.99)	0.33	-1.07 (-3.05, 0.91)	0.29

[a] Standard deviations for Shannon Index: 0.49, 0.56, 0.46, 0.48 for six-week, one-year, two-year, and three-year microbiomes respectively. Standard deviations for Simpson Index: 0.16, 0.09, 0.04, 0.03 for six-week, one-year, two-year, and three-year microbiomes respectively. Standard deviations for Taxa Count: 11.8, 35.1, 45.1, 67.6 for six-week, one-year, two-year, and three-year microbiomes respectively.

[b] Basic model adjusts for age at SRS-2, maternal education, marital status, maternal age, paternal age, and child sex

[c] Full model adjusts for basic model + maternal self-reported smoking during pregnancy, early exclusive breastfeeding, delivery mode, peripartum antibiotics, and gestational age

* indicates $p < 0.1$

Supplementary Table S3. Difference in Social Responsiveness Scale-2 total T-score per standard deviation[a] increase in within-subject diversity among term infants

Microbiome Age	Shannon				Simpson				Taxa Count			
	Basic Model[b]		Full Model[c]		Basic Model		Full Model		Basic Model		Full Model	
	Estimate (95% CI)	p	Estimate (95% CI)	p	Estimate (95% CI)	p	Estimate (95% CI)	p	Estimate (95% CI)	p	Estimate (95% CI)	p
Six Weeks	-0.24 (-0.98, 0.48)	0.51	-0.39 (-1.13, 0.34)	0.30	-0.65 (-0.99, 0.46)	0.47	-0.36 (-1.08, 0.35)	0.32	-0.44 (-1.17, 0.30)	0.25	-0.45 (-1.17, 0.28)	0.23
One Year	0.04 (-0.62, 0.70)	0.90	0.10 (-0.59, 0.79)	0.77	-0.24 (-0.90, 0.42)	0.48	-0.14 (-0.82, 0.54)	0.69	0.32 (-0.35,1)	0.35	0.24 (-0.46, 0.95)	0.50
Two Years	0.60 (-0.13, 1.33)	0.11	0.65 (-0.08, 1.37)	0.08*	0.28 (-0.46, 1.02)	0.46	0.33 (-0.41, 1.06)	0.39	0.51 (-0.23, 1.25)	0.18	0.53 (-0.22,1.28)	0.17
Three Years	-0.63 (-1.42, 0.16)	0.12	-0.63 (-1.42, 0.15)	0.12	-0.60 (-1.38, 0.19)	0.14	-0.57 (-1.35, 0.22)	0.16	-0.55 (-1.36, 0.25)	0.18	-0.58 (-1.37, 0.21)	0.16

[a] Standard deviations for Shannon Index: 0.49, 0.56, 0.44, 0.48 for six-week, one-year, two-year, and three-year microbiomes respectively. Standard deviations for Simpson Index: 0.16, 0.09, 0.04, 0.03 for six-week, one-year, two-year, and three-year microbiomes respectively. Standard deviations for Taxa Count: 11.5, 34.3, 42.9, 68.8 for six-week, one-year, two-year, and three-year microbiomes respectively.
[b] Basic model adjusts for age at SRS-2, maternal education, marital status, maternal age, paternal age, and child sex
[c] Full model adjusts for basic model + maternal self-reported smoking during pregnancy, early exclusive breastfeeding, delivery mode, peripartum antibiotics, and gestational age
* indicates p < 0.1

Supplementary Table S4. Difference in Social Responsiveness Scale-2 total T-score per standard deviation[a] increase in within-subject diversity adjusting for cotinine

Microbiome Age	Shannon				Simpson				Taxa Count			
	Basic Model[b]		Full Model[c]		Basic Model		Full Model		Basic Model		Full Model	
	Estimate (95% CI)	p	Estimate (95% CI)	p	Estimate (95% CI)	p	Estimate (95% CI)	p	Estimate (95% CI)	p	Estimate (95% CI)	p
Six Weeks	-0.23 (-1.03, 0.57)	0.57	-0.45 (-1.24, 0.34)	0.26	-0.28 (-1.07, 0.51)	0.48	-0.42 (-1.20, 0.35)	0.29	-0.24 (-1.04, 0.57)	0.56	-0.48 (-1.27, 0.30)	0.23
One Year	0.14 (-0.57, 0.85)	0.69	0.17 (-0.55, 0.90)	0.64	-0.06 (-0.77, 0.65)	0.87	-0.02 (-0.74, 0.70)	0.96	0.51 (-0.21, 1.23)	0.17	0.41 (-0.35, 1.17)	0.30
Two Years	-0.18 (-0.98, 0.62)	0.66	0.04 (-0.76, 0.85)	0.92	-0.81 (-1.60, -0.02)	**0.046**	-0.54 (-1.35, 0.26)	0.19	-0.01 (-0.82, 0.79)	0.97	0.01 (-0.81, 0.82)	0.98
Three Years	-0.52 (-1.33, 0.29)	0.21	-0.61 (-1.42, 0.20)	0.14	-0.45 (-1.25, 0.35)	0.27	-0.50 (-1.31, 0.30)	0.22	-0.55 (-1.37, 0.27)	0.19	-0.63 (-1.45, 0.19)	0.13

[a] Standard deviations for Shannon Index: 0.48, 0.55, 0.45, 0.48 for six-week, one-year, two-year, and three-year microbiomes respectively. Standard deviations for Simpson Index: 0.16, 0.08, 0.04, 0.03 for six-week, one-year, two-year, and three-year microbiomes respectively. Standard deviations for Taxa Count: 12, 35.6, 43.2, 67.9 for six-week, one-year, two-year, and three-year microbiomes respectively.
[b] Basic model adjusts for age at SRS-2, maternal education, marital status, maternal age, paternal age, and child sex
[c] Full model adjusts for basic model + maternal urinary cotinine during pregnancy, early exclusive breastfeeding, delivery mode, peripartum antibiotics, and gestational age
bold indicates p < 0.05

Supplementary Table S5. Difference in Social Responsiveness Scale-2 total T-score per standard deviation[a] increase in within-subject diversity adjusting for any breastfeeding at six months

Microbiome Age	Shannon				Simpson				Taxa Count			
	Basic Model[b]		Full Model[c]		Basic Model		Full Model		Basic Model		Full Model	
	Estimate (95% CI)	p	Estimate (95% CI)	p	Estimate (95% CI)	p	Estimate (95% CI)	p	Estimate (95% CI)	p	Estimate (95% CI)	p
Six Weeks[d]	-0.26 (-0.98, 0.46)	0.48	-0.38 (-1.09, 0.32)	0.29	-0.34 (-1.06, 0.37)	0.34	-0.43 (-1.12, 0.27)	0.23	-0.17 (-0.89, 0.55)	0.64	-0.24 (-0.94, 0.46)	0.50
One Year	0.07 (-0.64, 0.71)	0.92	0.08 (-0.62, 0.78)	0.83	-0.25 (-0.92, 0.42)	0.47	-0.16 (-0. 86, 0.53)	0.64	0.49 (-0.18, 1.17)	0.16	0.32 (-0.39, 1.04)	0.38
Two Years	-0.03 (-0.79, 0.74)	0.94	0.11 (-0.65, 0.87)	0.78	-0.67 (-1.42, 0.08)	0.08*	-0.46 (-1.22, 0.30)	0.24	0.14 (-0.62, 0.90)	0.72	0.11 (-0.66, 0.88)	0.78
Three Years	-0.48 (-1.23, 0.28)	0.22	-0.57 (-1.35, 0.21)	0.15	-0.43 (-1.18, 0.31)	0.26	-0.50 (-1.28, 0.27)	0.21	-0.44 (-1.20, 0.32)	0.26	-0.49 (-1.27, 0.29)	0.22

[a] Standard deviations for Shannon Index: 0.48, 0.56, 0.46, 0.48 for six-week, one-year, two-year, and three-year microbiomes respectively. Standard deviations for Simpson Index: 0.16, 0.09, 0.04, 0.03 for six-week, one-year, two-year, and three-year microbiomes respectively. Standard deviations for Taxa Count: 11.8, 35.2, 45, 67.8 for six-week, one-year, two-year, and three-year microbiomes respectively.

[b] Basic model adjusts for age at SRS-2, maternal education, marital status, maternal age, paternal age, and child sex and at six weeks includes any breastfeeding at six months

[c] Full model adjusts for basic model + maternal self-reported smoking during pregnancy, early exclusive breastfeeding, delivery mode, peripartum antibiotics, gestational age, and any breastfeeding at six months

[d] At six weeks the basic model additionally adjusts for any breastfeeding at six months

* indicates $p < 0.1$

Supplementary Table S6. Difference in Social Responsiveness Scale-2 total T-score per standard deviation[a] increase in within-subject diversity adjusting for the Parenting Relationship Questionnaire

Microbiome Age	Shannon				Simpson				Taxa Count			
	Basic Model[b]		Full Model[c]		Basic Model		Full Model		Basic Model		Full Model	
	Estimate (95% CI)	p	Estimate (95% CI)	p	Estimate (95% CI)	p	Estimate (95% CI)	p	Estimate (95% CI)	p	Estimate (95% CI)	p
Six Weeks	-0.06 (-0.72, 0.59)	0.59	-0.28 (-0.92, 0.37)	0.40	-0.08 (-0.73, 0.57)	0.81	-0.25 (-0.89, 0.38)	0.44	-0.28 (-0.93, 0.38)	0.41	-0.36 (-0.99, 0.28)	0.27
One Year	-0.04 (-0.65, 0.57)	0.90	0.003 (-0.61, 0.62)	0.99	-0.16 (-0.77, 0.45)	0.61	-0.06 (-0.67, 0.56)	0.85	0.52 (-0.15, 1.20)	0.13	0.11 (-0.53, 0.75)	0.74
Two Years	-0.09 (-0.78, 0.61)	0.80	0.07 (-0.62, 0.78)	0.85	-0.51 (-1.21, 0.18)	0.15	-0.34 (-1.04, 0.36)	0.34	-0.09 (-0.79, 0.61)	0.81	-0.03 (-0.73, 0.67)	0.93
Three Years	-0.28 (-1.00, 0.44)	0.45	-0.34 (-1.06, 0.38)	0.35	-0.23 (-0.94, 0.49)	0.54	-0.25 (-0.97, 0.46)	0.49	-0.26 (-0.98, 0.46)	0.49	-0.29 (-1.01, 0.43)	0.43

[a] Standard deviations for Shannon Index: 0.49, 0.56, 0.46, 0.48 for six-week, one-year, two-year, and three-year microbiomes respectively. Standard deviations for Simpson Index: 0.16, 0.09, 0.04, 0.03 for six-week, one-year, two-year, and three-year microbiomes respectively. Standard deviations for Taxa Count: 11.8, 35.2, 45.3, 68 for six-week, one-year, two-year, and three-year microbiomes respectively.

[b] Basic model adjusts for age at SRS-2, maternal education, marital status, maternal age, paternal age, child sex, and Parenting Relationship Questionnaire

[c] Full model adjusts for basic model + maternal self-reported smoking during pregnancy, early exclusive breastfeeding, delivery mode, peripartum antibiotics, and gestational age

Supplementary Table S7. Difference in Social Responsiveness Scale-2 total T-score per standard deviation[a] increase in within-subject diversity among subjects with microbiome sequencing at all time points

Microbiome Age	Shannon				Simpson				Taxa Count			
	Basic Model[b]		Full Model[c]		Basic Model		Full Model		Basic Model		Full Model	
	Estimate (95% CI)	p	Estimate (95% CI)	p	Estimate (95% CI)	p	Estimate (95% CI)	p	Estimate (95% CI)	p	Estimate (95% CI)	p
Six Weeks	-0.47 (-2.66, 1.73)	0.68	-0.09 (-3.02, 2.83)	0.95	-0.65 (-2.76, 1.46)	0.56	-0.45 (-3.43, 2.52)	0.77	-0.89 (-3.53, 1.75)	0.52	-1.01 (-4.32, 2.30)	0.57
One Year	0.74 (-1.34, 2.82)	0.50	0.05 (-3.06, 3.15)	0.98	0.48 (-1.83, 2.79)	0.69	-0.30 (-3.31, 2.72)	0.85	1.22 (-0.91, 3.35)	0.28	1.03 (-2.13, 4.20)	0.54
Two Years	0.29 (-2.31, 2.89)	0.83	0.13 (-3.44, 3.70)	0.94	0.09 (-2.99, 3.17)	0.96	0.44 (-4.01, 4.89)	0.85	0.48 (-1.81, 2.78)	0.69	-0.01 (-3.54, 3.51)	0.99
Three Years	-0.22 (-2.33, 1.89)	0.84	-2.38 (-5.39, 0.62)	0.16	-0.34 (-2.40, 1.72)	0.75	-1.80 (-4.38, 0.79)	0.22	0.61 (-1.70, 2.91)	0.62	-0.84 (-5.11, 3.43)	0.71

[a] Standard deviations for Shannon Index: 0.42, 0.5, 0.35, 0.45 for six-week, one-year, two-year, and three-year microbiomes respectively. Standard deviations for Simpson Index: 0.13, 0.09, 0.03, 0.02 for six-week, one-year, two-year, and three-year microbiomes respectively. Standard deviations for Taxa Count: 10.1, 29, 39.2, 60.5 for six-week, one-year, two-year, and three-year microbiomes respectively.

[b] Basic model adjusts for age at SRS-2, maternal education, marital status, maternal age, paternal age, and child sex

[c] Full model adjusts for basic model + maternal self-reported smoking during pregnancy, early exclusive breastfeeding, delivery mode, peripartum antibiotics, and gestational age

Supplementary Table S8. Difference in Social Responsiveness Scale-2 total T-score per standard deviation[a] increase in within-subject diversity calculated from metagenomic data

Microbiome Age	Shannon				Simpson				Taxa Count			
	Basic Model[b]		Full Model[c]		Basic Model		Full Model		Basic Model		Full Model	
	Estimate (95% CI)	p	Estimate (95% CI)	p	Estimate (95% CI)	p	Estimate (95% CI)	p	Estimate (95% CI)	p	Estimate (95% CI)	p
Six Weeks	-0.17 (-1.06, 0.72)	0.70	-0.18 (-1.10, 0.74)	0.70	-0.17 (-1.05, 0.72)	0.72	-0.14 (-1.05, 0.77)	0.76	0.53 (-0.38, 1.45)	0.26	0.39 (-0.54, 1.31)	0.41
One Year	-0.13 (-1.03, 0.76)	0.77	-0.29 (-1.18, 0.60)	0.52	-0.19 (-1.09, 0.71)	0.68	-0.29 (-1.18, 0.61)	0.53	-0.03 (-0.90, 0.85)	0.95	-0.34 (-1.24, 0.56)	0.46

[a] Standard deviations for Shannon Index: 0.58, 0.46 for six-week and one-year microbiomes respectively. Standard deviations for Simpson Index: 0.22 0.1 for six-week and one-year microbiomes respectively. Standard deviations for Taxa Count: 11.8, 15.7 for six-week and one-year microbiomes respectively.

[b] Basic model adjusts for age at SRS-2, maternal education, marital status, maternal age, paternal age, and child sex

[c] Full model adjusts for basic model + maternal self-reported smoking during pregnancy, early exclusive breastfeeding, delivery mode, peripartum antibiotics, and gestational age

Supplementary Table S9. Marginal p-values for association between GUniFrac distances and total T-scores on SRS-2

Microbiome Age	Basic Model[a]	Full Model[b]
Six Weeks	0.97	0.99
One Year	0.01	0.06
Two Years	0.77	0.96
Three Years	0.41	0.35

[a] Basic model adjusts for age at SRS2, maternal education, marital status, maternal age, paternal age, and child sex

[b] Full model adjusts for basic model + maternal self-reported smoking during pregnancy, early exclusive breastfeeding, delivery mode, peripartum antibiotics, and gestational age

Supplementary Table S10. Marginal p-values for association between GUniFrac distances and total T-scores on the SRS-2 at three years, sensitivity analyses

Modification	Microbiome Age	Basic Model	Full Model
SRS-2 Raw Scores[a]	Six Weeks	0.34	0.34
	One Year	0.007	0.04
	Two Years	0.74	0.95
	Three Years	0.40	0.32
Among Term Infants[a]	Six Weeks	0.98	0.95
	One Year	0.07	0.19
	Two Years	0.62	0.48
	Three Years	0.40	0.28
Adjusting for cotinine in place of self-report of smoking[b]	Six Weeks	0.96	0.99
	One Year	0.02	0.10
	Two Years	0.74	0.92
	Three Years	0.44	0.41
Adjusting for breastfeeding at six months[c]	Six Weeks	0.97	0.98
	One Year	0.006	0.048
	Two Years	0.77	0.95
	Three Years	0.65	0.64
Adjusting for PRQ[d]	Six Weeks	0.85	0.91
	One Year	0.05	0.29
	Two Years	0.45	0.86
	Three Years	0.50	0.37
Among subjects with samples at all time points[a]	Six Weeks	0.88	0.88
	One Year	0.21	0.52
	Two Years	0.27	0.18
	Three Years	0.03	0.11

[a] Covariates in basic model: age at SRS-2, maternal education, marital status, maternal age, paternal age, child sex; covariates in full model: basic + maternal self-reported smoking during pregnancy, early exclusive breastfeeding, delivery mode, peripartum antibiotics, and gestational age

[b] Covariates the same as [a] except full model adjusts for maternal urinary cotinine during pregnancy in place of maternal self-report of smoking

[c] Covariates the same as [a] with the addition of an indicator variable for any breastfeeding at six months in the basic and full models for six-week microbiome analyses and in the full model for all other microbiome age analyses

[d] Covariates the same as [a] with the addition of the Parenting Relationship Questionnaire (PRQ)

Supplementary Table S11. Taxa associated with total T-scores on the Social Responsiveness Scale-2 in primary analyses ($p < 0.05$)

Phylum	Class	Order	Family	Genus	Species	Estimate[a]	SE[b]	N[c]	N not 0[d]	pval	Model[e]	MB Age[f]
Actinobacteria	Actinobacteria	Bifidobacteriales	Bifidobacteriaceae	Bifidobacterium	adolescentis	0.36	0.16	129	49	0.03	Full	2 Y
Actinobacteria	Actinobacteria	Bifidobacteriales	Bifidobacteriaceae	Bifidobacterium	adolescentis	0.36	0.16	129	49	0.02	Basic	2 Y
Actinobacteria	Actinobacteria	Bifidobacteriales	Bifidobacteriaceae	Bifidobacterium		1.02	0.43	129	128	0.02	Full	2 Y
Actinobacteria	Actinobacteria	Bifidobacteriales	Bifidobacteriaceae	Bifidobacterium		1.28	0.44	129	128	0.00	Basic	2 Y
Actinobacteria	Actinobacteria	Bifidobacteriales	Bifidobacteriaceae	Bifidobacterium		-0.70	0.31	129	93	0.02	Full	2 Y
Actinobacteria	Actinobacteria	Bifidobacteriales	Bifidobacteriaceae	Bifidobacterium		-0.59	0.30	129	93	0.04	Basic	2 Y
Actinobacteria	Actinobacteria	Bifidobacteriales	Bifidobacteriaceae	Bifidobacterium		0.04	0.01	129	18	0.00	Full	2 Y
Actinobacteria	Actinobacteria	Bifidobacteriales	Bifidobacteriaceae	Bifidobacterium		0.05	0.01	129	18	8.26 E-05	Basic	2 Y
Actinobacteria	Coriobacteria	Coriobacteriales	Coriobacteriaceae	Collinsella	aerofaciens	0.21	0.09	129	73	0.02	Full	2 Y
Actinobacteria	Coriobacteria	Coriobacteriales	Coriobacteriaceae	Collinsella	aerofaciens	0.25	0.09	129	73	0.00	Basic	2 Y
Actinobacteria	Coriobacteria	Coriobacteriales	Coriobacteriaceae	Collinsella	aerofaciens	0.11	0.05	140	89	0.03	Full	3 Y
Actinobacteria	Coriobacteria	Coriobacteriales	Coriobacteriaceae	Collinsella	aerofaciens	0.11	0.04	140	89	0.02	Basic	3 Y
Bacteroidetes	Bacteroidia	Bacteroidales	Bacteroidaceae	Bacteroides	caccae	0.25	0.08	140	87	0.00	Full	3 Y
Bacteroidetes	Bacteroidia	Bacteroidales	Bacteroidaceae	Bacteroides	caccae	0.23	0.08	140	87	0.00	Basic	3 Y
Bacteroidetes	Bacteroidia	Bacteroidales	Bacteroidaceae	Bacteroides	caccae	-0.20	0.08	129	34	0.01	Full	2 Y
Bacteroidetes	Bacteroidia	Bacteroidales	Bacteroidaceae	Bacteroides	uniformis	-0.53	0.21	158	58	0.01	Full	1 Y
Bacteroidetes	Bacteroidia	Bacteroidales	Bacteroidaceae	Bacteroides	uniformis	-0.47	0.20	158	58	0.02	Basic	1 Y
Bacteroidetes	Bacteroidia	Bacteroidales	Bacteroidaceae	Bacteroides		-0.64	0.28	166	48	0.02	Full	6 W
Bacteroidetes	Bacteroidia	Bacteroidales	Bacteroidaceae	Bacteroides		-0.55	0.26	166	48	0.03	Basic	6 W
Bacteroidetes	Bacteroidia	Bacteroidales	Porphyromonadaceae	Parabacteroides		0.09	0.04	140	90	0.04	Full	3 Y
Bacteroidetes	Bacteroidia	Bacteroidales	Prevotellaceae	Prevotella	copri	-0.00	0.00	166	17	0.04	Full	6 W
Bacteroidetes	Bacteroidia	Bacteroidales	Prevotellaceae	Prevotella	copri	-0.67	0.24	158	32	0.00	Full	1 Y
Bacteroidetes	Bacteroidia	Bacteroidales	Prevotellaceae	Prevotella	copri	-0.68	0.23	158	32	0.00	Basic	1 Y
Firmicutes	Bacilli	Lactobacillales	Enterococcaceae	Enterococcus		-0.05	0.02	140	17	0.04	Full	3 Y
Firmicutes	Bacilli	Lactobacillales	Enterococcaceae	Enterococcus		0.28	0.13	140	23	0.03	Full	3 Y
Firmicutes	Bacilli	Lactobacillales	Enterococcaceae	Enterococcus		0.30	0.13	140	23	0.02	Basic	3 Y
Firmicutes	Bacilli	Lactobacillales	Lactobacillaceae	Lactobacillus	zeae	-0.04	0.01	140	26	0.01	Full	3 Y
Firmicutes	Bacilli	Lactobacillales	Streptococcaceae	Streptococcus		0.01	0.00	129	13	0.02	Full	2 Y
Firmicutes	Bacilli	Lactobacillales	Streptococcaceae	Streptococcus		0.00	0.00	129	13	0.03	Basic	2 Y
Firmicutes	Bacilli	Lactobacillales	Streptococcaceae	Streptococcus		-0.11	0.04	166	96	0.01	Full	6 W
Firmicutes	Bacilli	Lactobacillales	Streptococcaceae	Streptococcus		-0.11	0.04	166	96	0.01	Basic	6 W
Firmicutes	Bacilli	Lactobacillales	Streptococcaceae	Streptococcus		0.47	0.22	166	35	0.03	Basic	6 W
Firmicutes	Clostridia	Clostridiales	[Tissierellaceae]	Anaerococcus		0.00	0.00	140	23	0.04	Full	3 Y
Firmicutes	Clostridia	Clostridiales	[Tissierellaceae]	Anaerococcus		0.00	0.00	140	23	0.03	Basic	3 Y
Firmicutes	Clostridia	Clostridiales	Christensenellaceae			0.00	0.00	140	35	0.02	Full	3 Y
Firmicutes	Clostridia	Clostridiales	Christensenellaceae			0.00	0.00	140	35	0.02	Basic	3 Y
Firmicutes	Clostridia	Clostridiales	Clostridiaceae	Clostridium	perfringens	1.08	0.51	166	61	0.03	Basic	6 W
Firmicutes	Clostridia	Clostridiales	Clostridiaceae	Clostridium		-0.25	0.11	140	118	0.02	Full	3 Y

Phylum	Class	Order	Family	Genus	Species	Estimate[a]	SE[b]	N[c]	N not 0[d]	pval	Model[e]	MB Age[f]
Firmicutes	Clostridia	Clostridiales	Eubacteriaceae	Anaerofustis		0.00	0.00	129	15	0.00	Full	2 Y
Firmicutes	Clostridia	Clostridiales	Eubacteriaceae	Anaerofustis		0.00	0.00	129	15	0.01	Basic	2 Y
Firmicutes	Clostridia	Clostridiales	Eubacteriaceae	Anaerofustis		0.00	0.00	140	37	0.00	Full	3 Y
Firmicutes	Clostridia	Clostridiales	Eubacteriaceae	Anaerofustis		0.00	0.00	140	37	0.00	Basic	3 Y
Firmicutes	Clostridia	Clostridiales	Eubacteriaceae	Pseudoramibacter _Eubacterium		0.04	0.02	158	45	0.02	Full	1 Y
Firmicutes	Clostridia	Clostridiales	Eubacteriaceae	Pseudoramibacter _Eubacterium		0.04	0.01	158	45	0.01	Basic	1 Y
Firmicutes	Clostridia	Clostridiales	Lachnospiraceae	[Ruminococcus]	gnavus	-1.02	0.45	166	64	0.02	Full	6 W
Firmicutes	Clostridia	Clostridiales	Lachnospiraceae	[Ruminococcus]	gnavus	-0.97	0.42	166	64	0.02	Basic	6 W
Firmicutes	Clostridia	Clostridiales	Lachnospiraceae	[Ruminococcus]	gnavus	0.13	0.04	129	96	0.00	Full	2 Y
Firmicutes	Clostridia	Clostridiales	Lachnospiraceae	[Ruminococcus]	gnavus	0.17	0.04	129	96	0.00	Basic	2 Y
Firmicutes	Clostridia	Clostridiales	Lachnospiraceae	[Ruminococcus]	torques	0.05	0.02	158	51	0.02	Full	1 Y
Firmicutes	Clostridia	Clostridiales	Lachnospiraceae	[Ruminococcus]	torques	0.06	0.02	158	51	0.00	Basic	1 Y
Firmicutes	Clostridia	Clostridiales	Lachnospiraceae	[Ruminococcus]		0.22	0.09	158	92	0.02	Basic	1 Y
Firmicutes	Clostridia	Clostridiales	Lachnospiraceae	Blautia	obeum	0.19	0.09	158	32	0.04	Full	1 Y
Firmicutes	Clostridia	Clostridiales	Lachnospiraceae	Blautia	producta	0.04	0.01	158	41	0.00	Full	1 Y
Firmicutes	Clostridia	Clostridiales	Lachnospiraceae	Blautia	producta	0.04	0.01	158	41	0.00	Basic	1 Y
Firmicutes	Clostridia	Clostridiales	Lachnospiraceae	Blautia	producta	0.28	0.06	158	17	6.57 E-05	Full	1 Y
Firmicutes	Clostridia	Clostridiales	Lachnospiraceae	Blautia	producta	0.34	0.07	158	17	5.54 E-06	Basic	1 Y
Firmicutes	Clostridia	Clostridiales	Lachnospiraceae	Blautia		0.06	0.02	129	77	0.01	Full	2 Y
Firmicutes	Clostridia	Clostridiales	Lachnospiraceae	Blautia		-0.00	0.00	140	29	0.04	Full	3 Y
Firmicutes	Clostridia	Clostridiales	Lachnospiraceae	Blautia		0.03	0.01	129	16	0.04	Full	2 Y
Firmicutes	Clostridia	Clostridiales	Lachnospiraceae	Blautia		0.03	0.01	129	16	0.02	Basic	2 Y
Firmicutes	Clostridia	Clostridiales	Lachnospiraceae	Blautia		0.29	0.10	129	91	0.00	Full	2 Y
Firmicutes	Clostridia	Clostridiales	Lachnospiraceae	Blautia		0.23	0.10	129	91	0.02	Basic	2 Y
Firmicutes	Clostridia	Clostridiales	Lachnospiraceae	Blautia		-0.05	0.02	140	82	0.03	Full	3 Y
Firmicutes	Clostridia	Clostridiales	Lachnospiraceae	Blautia		0.00	0.00	140	27	0.04	Basic	3 Y
Firmicutes	Clostridia	Clostridiales	Lachnospiraceae	Blautia		0.00	0.00	129	23	0.04	Full	2 Y
Firmicutes	Clostridia	Clostridiales	Lachnospiraceae	Clostridium	citroniae	0.02	0.01	140	105	0.04	Full	3 Y
Firmicutes	Clostridia	Clostridiales	Lachnospiraceae	Coprococcus		0.05	0.01	61	61	0.00	Full	2 Y
Firmicutes	Clostridia	Clostridiales	Lachnospiraceae	Coprococcus		0.06	0.01	61	61	9.00 E-05	Basic	2 Y
Firmicutes	Clostridia	Clostridiales	Lachnospiraceae	Coprococcus		0.03	0.01	22	22	0.02	Full	3 Y
Firmicutes	Clostridia	Clostridiales	Lachnospiraceae	Coprococcus		0.05	0.01	22	22	0.00	Basic	3 Y
Firmicutes	Clostridia	Clostridiales	Lachnospiraceae	Coprococcus		0.02	0.00	14	14	0.00	Full	2 Y
Firmicutes	Clostridia	Clostridiales	Lachnospiraceae	Coprococcus		0.01	0.00	14	14	0.01	Basic	2 Y
Firmicutes	Clostridia	Clostridiales	Lachnospiraceae	Coprococcus		-0.01	0.00	24	24	0.00	Full	3 Y

Phylum	Class	Order	Family	Genus	Species	Estimate[a]	SE[b]	N[c]	N not 0[d]	pval	Model[e]	MB Age[f]
Firmicutes	Clostridia	Clostridiales	Lachnospiraceae	Coprococcus		-0.01	0.00	24	24	0.01	Basic	3 Y
Firmicutes	Clostridia	Clostridiales	Lachnospiraceae	Dorea	formicigenerans	0.06	0.03	25	25	0.04	Full	1 Y
Firmicutes	Clostridia	Clostridiales	Lachnospiraceae	Dorea		-0.21	0.08	88	88	0.01	Full	2 Y
Firmicutes	Clostridia	Clostridiales	Lachnospiraceae	Lachnospira		0.00	0.00	16	16	0.00	Full	2 Y
Firmicutes	Clostridia	Clostridiales	Lachnospiraceae	Lachnospira		0.00	0.00	16	16	0.00	Basic	2 Y
Firmicutes	Clostridia	Clostridiales	Lachnospiraceae	Roseburia		-0.06	0.02	90	90	0.01	Full	3 Y
Firmicutes	Clostridia	Clostridiales	Lachnospiraceae	Roseburia		-0.07	0.02	90	90	0.00	Basic	3 Y
Firmicutes	Clostridia	Clostridiales	Lachnospiraceae			0.00	0.00	15	15	0.04	Full	2 Y
Firmicutes	Clostridia	Clostridiales	Lachnospiraceae			-0.00	0.00	24	24	0.02	Full	3 Y
Firmicutes	Clostridia	Clostridiales	Lachnospiraceae			-0.00	0.00	24	24	0.02	Basic	3 Y
Firmicutes	Clostridia	Clostridiales	Lachnospiraceae			0.00	0.00	14	14	0.04	Full	2 Y
Firmicutes	Clostridia	Clostridiales	Lachnospiraceae			-0.03	0.01	28	28	0.03	Basic	2 Y
Firmicutes	Clostridia	Clostridiales	Lachnospiraceae			0.00	0.00	15	15	0.01	Full	3 Y
Firmicutes	Clostridia	Clostridiales	Lachnospiraceae			0.00	0.00	15	15	0.00	Basic	3 Y
Firmicutes	Clostridia	Clostridiales	Lachnospiraceae			-0.00	0.00	18	18	0.04	Full	2 Y
Firmicutes	Clostridia	Clostridiales	Lachnospiraceae			0.01	0.00	27	27	0.02	Basic	2 Y
Firmicutes	Clostridia	Clostridiales	Lachnospiraceae			0.00	0.00	16	16	0.00	Full	1 Y
Firmicutes	Clostridia	Clostridiales	Lachnospiraceae			0.01	0.00	158	16	0.00	Basic	1 Y
Firmicutes	Clostridia	Clostridiales	Peptostreptococcaceae			-0.01	0.00	140	27	0.04	Basic	3 Y
Firmicutes	Clostridia	Clostridiales	Peptostreptococcaceae			-0.23	0.09	140	126	0.01	Full	3 Y
Firmicutes	Clostridia	Clostridiales	Peptostreptococcaceae			-0.22	0.09	140	126	0.01	Basic	3 Y
Firmicutes	Clostridia	Clostridiales	Ruminococcaceae	Butyricicoccus	pullicaecorum	0.00	0.00	158	40	0.04	Basic	1 Y
Firmicutes	Clostridia	Clostridiales	Ruminococcaceae	Butyricicoccus	pullicaecorum	0.01	0.00	140	108	0.00	Full	3 Y
Firmicutes	Clostridia	Clostridiales	Ruminococcaceae	Butyricicoccus	pullicaecorum	0.00	0.00	140	108	0.00	Basic	3 Y
Firmicutes	Clostridia	Clostridiales	Ruminococcaceae	Faecalibacterium	prausnitzii	-0.32	0.16	140	85	0.04	Basic	3 Y
Firmicutes	Clostridia	Clostridiales	Ruminococcaceae	Oscillospira		-0.00	0.00	129	32	0.03	Full	2 Y
Firmicutes	Clostridia	Clostridiales	Ruminococcaceae	Oscillospira		-0.00	0.00	129	32	0.02	Basic	2 Y
Firmicutes	Clostridia	Clostridiales	Ruminococcaceae	Oscillospira		0.29	0.14	166	34	0.04	Full	6 W
Firmicutes	Clostridia	Clostridiales	Ruminococcaceae	Oscillospira		0.42	0.14	166	34	0.00	Basic	6 W
Firmicutes	Clostridia	Clostridiales	Ruminococcaceae	Oscillospira		0.17	0.08	129	47	0.04	Basic	2 Y
Firmicutes	Clostridia	Clostridiales	Ruminococcaceae	Oscillospira		0.04	0.01	158	28	0.01	Full	1 Y
Firmicutes	Clostridia	Clostridiales	Ruminococcaceae	Oscillospira		0.05	0.01	158	28	0.00	Basic	1 Y
Firmicutes	Clostridia	Clostridiales	Ruminococcaceae	Ruminococcus		-0.00	0.00	129	16	0.01	Full	2 Y
Firmicutes	Clostridia	Clostridiales	Ruminococcaceae	Ruminococcus		0.27	0.13	140	106	0.04	Basic	3 Y
Firmicutes	Clostridia	Clostridiales	Ruminococcaceae	Ruminococcus		0.00	0.00	129	14	0.03	Full	2 Y
Firmicutes	Clostridia	Clostridiales	Ruminococcaceae	Ruminococcus		0.00	0.00	129	14	0.03	Basic	2 Y
Firmicutes	Clostridia	Clostridiales	Ruminococcaceae	Ruminococcus		0.02	0.00	129	14	0.01	Full	2 Y
Firmicutes	Clostridia	Clostridiales	Ruminococcaceae	Ruminococcus		0.02	0.00	129	14	0.00	Basic	2 Y
Firmicutes	Clostridia	Clostridiales	Veillonellaceae	Megaphaera		-0.05	0.02	158	40	0.02	Basic	1 Y
Firmicutes	Clostridia	Clostridiales	Veillonellaceae	Veillonella	dispar	-0.25	0.10	158	100	0.01	Full	1 Y

Phylum	Class	Order	Family	Genus	Species	Estimate[a]	SE[b]	N[c]	N not 0[d]	pval	Model[e]	MB Age[f]
Firmicutes	Clostridia	Clostridiales	Veillonellaceae	Veillonella	dispar	-0.26	0.10	158	100	0.01	Basic	1 Y
Firmicutes	Clostridia	Clostridiales				0.03	0.01	129	24	0.01	Full	2 Y
Firmicutes	Clostridia	Clostridiales				0.03	0.01	129	24	0.01	Basic	2 Y
Firmicutes	Erysipelotrichi	Erysipelotrichales	Erysipelotrichaceae	[Eubacterium]	dolichum	-0.00	0.00	140	17	0.01	Full	3 Y
Firmicutes	Erysipelotrichi	Erysipelotrichales	Erysipelotrichaceae	[Eubacterium]	dolichum	-0.00	0.00	140	17	0.01	Basic	3 Y
Firmicutes	Erysipelotrichi	Erysipelotrichales	Erysipelotrichaceae	Coprobacillus		-0.00	0.00	129	45	0.00	Full	2 Y
Firmicutes	Erysipelotrichi	Erysipelotrichales	Erysipelotrichaceae	Coprobacillus		-0.00	0.00	129	45	0.01	Basic	2 Y
Firmicutes	Erysipelotrichi	Erysipelotrichales	Erysipelotrichaceae			0.30	0.11	158	18	0.01	Full	1 Y
Firmicutes	Erysipelotrichi	Erysipelotrichales	Erysipelotrichaceae			0.25	0.11	158	18	0.02	Basic	1 Y
Proteobacteria	Betaproteobacteria	Burkholderiales	Alcaligenaceae	Sutterella		0.01	0.00	129	37	0.04	Full	2 Y
Proteobacteria	Betaproteobacteria	Burkholderiales	Alcaligenaceae	Sutterella		-0.03	0.01	129	22	0.03	Basic	2 Y
Proteobacteria	Betaproteobacteria	Burkholderiales	Alcaligenaceae	Sutterella		0.00	0.00	129	15	0.00	Full	2 Y
Proteobacteria	Betaproteobacteria	Burkholderiales	Alcaligenaceae	Sutterella		0.00	0.00	129	15	0.02	Basic	2 Y
Proteobacteria	Betaproteobacteria	Burkholderiales	Comamonadaceae			0.00	0.00	129	37	0.01	Full	2 Y
Proteobacteria	Betaproteobacteria	Burkholderiales	Comamonadaceae			0.00	0.00	129	37	0.03	Basic	2 Y
Proteobacteria	Gammaproteobacteria	Enterobacteriales	Enterobacteriaceae	Erwinia		-0.03	0.01	166	51	0.00	Full	6 W
Proteobacteria	Gammaproteobacteria	Pasteurellales	Pasteurellaceae	Haemophilus	parainfluenzae	-0.00	0.00	158	22	0.04	Full	1 Y
Proteobacteria	Gammaproteobacteria	Pasteurellales	Pasteurellaceae	Haemophilus	parainfluenzae	-0.00	0.00	158	22	0.02	Basic	1 Y
Proteobacteria	Gammaproteobacteria	Pseudomonad-ales	Pseudomonadaceae	Pseudomonas		-0.00	0.00	158	16	0.04	Full	1 Y
Verrucomicrobia	Verrucomicrobiae	Verrucomicrobiales	Verrucomicrobiaceae	Akkermansia	muciniphila	0.07	0.02	129	23	0.00	Full	2 Y
Verrucomicrobia	Verrucomicrobiae	Verrucomicrobiales	Verrucomicrobiaceae	Akkermansia	muciniphila	0.05	0.02	129	23	0.04	Basic	2 Y
Verrucomicrobia	Verrucomicrobiae	Verrucomicrobiales	Verrucomicrobiaceae	Akkermansia	muciniphila	0.12	0.05	140	38	0.04	Full	3 Y

[a] MaAsLin models the microbiome as the outcome. Thus, estimate is increase in % relative abundance per point increase on SRS2 T-score
[b] SE: standard error
[c] Number of subjects in analysis
[d] Number of subjects with taxon present and sequenced
[e] Covariates in basic model: age at SRS-2, maternal education, marital status, maternal age, paternal age, child sex; covariates in full model: basic + maternal self-reported smoking during pregnancy, early exclusive breastfeeding, delivery mode, peripartum antibiotics, and gestational age
[f] MB Age: age of microbiome sample; 6W: six weeks; 1Y: one year; 2Y: two years; 3Y: three years

Supplementary Table S12. Taxa associated with Social Responsiveness Scale-2 (SRS-2) total T-scores at three years, sensitivity analyses (16S sequencing)

Modification	Microbiome Age	Family	Genus	Species	Estimate in Basic Model[a]	Estimate in Full Model[a]	Average Relative Abundance (%)[b]
SRS-2 Raw Scores[c]	One Year	Lachnospiraceae	Blautia	producta	0.36	0.30	0.10
		Lachnospiraceae			0.01	–	0.01
		Lachnospiraceae	Blautia	producta	-0.01	–	0.06
	Two Years	Lachnospiraceae	Coprococcus		0.07	0.06	0.05
		Lachnospiraceae	[Ruminococcus]	gnavus	0.18	0.14	0.2
		Bifidobacteriaceae	Bifidobacterium		0.06	0.05	0.03
	Three Years	Ruminococcaceae	Butyricicoccus	pullicaecorum	0.01	0.01	0.02
Among Term Infants[c]	One Year	Lachnospiraceae	Blautia	producta	0.48	–	0.02
	Three Years	Ruminococcaceae	Butyricicoccus	pullicaecorum	0.01	0.01	0.01
Adjusting for cotinine in place of self-report of smoking[d]	One Year	Lachnospiraceae	Blautia	producta	0.36	0.30	0.10
		Lachnospiraceae	Blautia	producta	0.15	0.11	0.07
		Lachnospiraceae			0.01	–	0.01
	Two Years	Lachnospiraceae	Coprococcus		0.07	–	0.06
		Lachnospiraceae	[Ruminococcus]	gnavus	0.17	–	0.21
		Bifidobacteriaceae	Bifidobacterium		0.06	0.05	0.03
		Lachnospiraceae	Blautia		–	0.35	0.86
		Alcaligenaceae	Sutterella		–	0.01	0.01
	Three Years	Ruminococcaceae	Butyricicoccus	pullicaecorum	0.01	0.01	0.02
Adjusting for breastfeeding at six months[e]	One Year	Lachnospiraceae	Blautia	producta	0.34	0.28	0.10
		Lachnospiraceae			0.01	–	0.01
	Two Years	Lachnospiraceae	Coprococcus		0.06	0.07	0.05
		Bifidobacteriaceae	Bifidobacterium		0.04	0.06	0.03
		Alcaligenaceae	Sutterella		0.01	–	0.01
		Ruminococcaceae			0.003	0.003	0.004
		Verrucomicrobiaceae	Akkermansia	muciniphila	0.08	–	0.07
		Lachnospiraceae	[Ruminococcus]	gnavus	–	0.17	0.21
		Bifidobacteriaceae	Bifidobacterium		–	1.41	2.94

Modification	Microbiome Age	Family	Genus	Species	Estimate in Basic Model[a]	Estimate in Full Model[a]	Average Relative Abundance (%)[b]
Adjusting for PRQ[f]	One Year	Lachnospiraceae	Blautia	producta	0.31	–	0.1
	Two Years	Lachnospiraceae	Coprococcus		0.06	–	0.05
		Bifidobacteriaceae	Bifidobacterium		0.05	–	0.03
		Ruminococcaceae	Ruminococcus		–	0.03	0.02
	Three Years	Ruminococcaceae	Butyricicoccus	pullicaecorum	0.01	0.01	0.02
		Lactobacillaceae	Lactobacillus	zeae	–	-0.07	0.05
Among subjects with samples at all time points[c]	One Year	Bacteroidaceae	Bacteroides	uniformis	–	-0.55	0.24
	Three Years	Ruminococcaceae	Ruminococcus		–	-0.01	0.003
		Ruminococcaceae	Oscillospira		–	-0.15	0.08
		Lachnospiraceae	Lachnospira		–	-0.09	0.04
		Ruminococcaceae			–	-0.004	0.003
		Clostridiaceae	Sarcina		–	-0.001	0.001
		Lachnospiraceae	Blautia		–	-0.04	0.02

[a] MaAsLin models the microbiome as the outcome. Thus, estimate is increase in % relative abundance per point increase on SRS2 T-score
[b] Mean relative abundance of specific amplicon sequence variant given microbiome age
[c] Covariates in basic model: age at SRS-2, maternal education, marital status, maternal age, paternal age, child sex; covariates in full model: basic + maternal self-reported smoking during pregnancy, early exclusive breastfeeding, delivery mode, peripartum antibiotics, and gestational age
[d] Covariates the same as [c] except full model adjusts for maternal urinary cotinine during pregnancy in place of maternal self-report of smoking
[e] Covariates the same as [c] with the addition of an indicator variable for any breastfeeding at six months in the basic and full models for six-week microbiome analyses and in the full model for all other microbiome age analyses
[f] Covariates the same as [c] with the addition of the Parenting Relationship Questionnaire (PRQ)

Supplementary Table S13. Association between Bacteroidetes:Firmicutes (B:F) ratio and total T-scores on the Social Responsiveness Scale-2

Microbiome Age	Median (IQR[a]) B:F Ratio	Basic Model[b]		Full Model[c]	
		Estimate (95%CI)	p	Estimate (95%CI)	p
Six Weeks	0.02 (1.85)	-0.01 (-0.03, 0.01)	0.20	-0.01 (-0.03, 0.01)	0.34
One Year	0.22 (0.53)	-0.42 (-1.29, 0.46)	0.36	-0.34 (-1.21, 0.53)	0.44
2 Years	0.13 (0.23)	-0.67 (-3.04, 1.70)	0.58	0.12 (-2.30, 2.55)	0.92
Three Years	0.16 (0.29)	1.08 (-1.88, 4.03)	0.48	1.25 (-1.73, 4.23)	0.41

[a] IQR = Interquartile range = 75th percentile – 25th percentile
[b] Basic model adjusts for age at SRS-2, maternal education, marital status, maternal age, paternal age, and child sex
[c] Full model adjusts for basic model + maternal self-reported smoking during pregnancy, early exclusive breastfeeding, delivery mode, peripartum antibiotics, and gestational age

Supplementary Table S14. Taxa associated with total T-scores on the Social Responsiveness Scale-2 in metagenomic analyses (p <0.05)

Phylum	Class	Order	Family	Genus	Species	Estimate[a]	SE[b]	N[c]	N not 0[d]	pval	Model[e]	MB Age[f]
Actinobacteria	Actinobacteria	Actinomycetales	Actinomycetaceae	Varibaculum	cambriense	0.000	0.000	101	17	0.02	Basic	6 W
Actinobacteria	Actinobacteria	Actinomycetales	Actinomycetaceae	Varibaculum	cambriense	0.000	0.000	101	17	0.01	Full	6 W
Actinobacteria	Actinobacteria	Actinomycetales	Corynebacteriaceae	Corynebacterium	pseudodiptheriticum	0.000	0.000	101	20	0.04	Full	6 W
Actinobacteria	Actinobacteria	Bifidobacteriales	Bifidobacteriaceae	Bifidobacterium	animalis	0.000	0.000	103	36	0.01	Basic	1 Y
Actinobacteria	Actinobacteria	Bifidobacteriales	Bifidobacteriaceae	Bifidobacterium	animalis	0.000	0.000	103	36	0.02	Full	1 Y
Actinobacteria	Actinobacteria	Coriobacteriales	Coriobacteriaceae	Adlercreutzia	equolifaciens	0.002	0.000	103	16	0.00	Basic	1 Y
Actinobacteria	Actinobacteria	Coriobacteriales	Coriobacteriaceae	Adlercreutzia	equolifaciens	0.002	0.000	103	16	0.00	Full	1 Y
Actinobacteria	Actinobacteria	Coriobacteriales	Coriobacteriaceae	Eggerthella	unclassified	0.003	0.001	103	97	0.04	Basic	1 Y
Actinobacteria	Actinobacteria	Coriobacteriales	Coriobacteriaceae	Eggerthella	unclassified	0.003	0.001	103	97	0.04	Full	1 Y
Bacteroidetes	Bacteroidia	Bacteroidales	Bacteroidaceae	Bacteroides	fragilis	-0.011	0.005	103	55	0.05	Basic	1 Y
Bacteroidetes	Bacteroidia	Bacteroidales	Bacteroidaceae	Bacteroides	xylanisolvens	-0.001	0.000	103	31	0.02	Full	1 Y
Firmicutes	Clostridia	Clostridiales	Eubacteriaceae	Anaerofustis	stercorihominis	0.001	0.000	103	13	0.01	Basic	1 Y
Firmicutes	Clostridia	Clostridiales	Eubacteriaceae	Anaerofustis	stercorihominis	0.000	0.000	103	13	0.04	Full	1 Y
Firmicutes	Clostridia	Clostridiales	Eubacteriaceae	Eubacterium	limosum	0.001	0.000	103	37	0.01	Basic	1 Y
Firmicutes	Clostridia	Clostridiales	Eubacteriaceae	Eubacterium	limosum	0.001	0.000	103	37	0.03	Full	1 Y
Firmicutes	Clostridia	Clostridiales	Lachnospiraceae	Dorea	formicigenerans	0.002	0.001	103	17	0.05	Basic	1 Y
Firmicutes	Clostridia	Clostridiales	Lachnospiraceae	Dorea	formicigenerans	0.003	0.001	103	17	0.02	Full	1 Y
Firmicutes	Clostridia	Clostridiales	Lachnospiraceae	Ruminococcus	gnavus	0.017	0.007	103	101	0.01	Basic	1 Y
Firmicutes	Clostridia	Clostridiales	Lachnospiraceae	Ruminococcus	gnavus	0.014	0.007	103	101	0.04	Full	1 Y
Firmicutes	Clostridia	Clostridiales	Lachnospiraceae	Ruminococcus	torques	0.008	0.002	103	100	0.00	Basic	1 Y
Firmicutes	Clostridia	Clostridiales	Lachnospiraceae	Ruminococcus	torques	0.008	0.002	103	100	0.00	Full	1 Y
Firmicutes	Clostridia	Clostridiales	Lachnospiraceae		bacterium 6_1_63FAA	0.001	0.000	103	16	0.00	Basic	1 Y
Firmicutes	Clostridia	Clostridiales	Lachnospiraceae		bacterium 6_1_63FAA	0.001	0.000	103	16	0.00	Full	1 Y
Firmicutes	Clostridia	Clostridiales		Flavonifractor	plautii	0.002	0.001	101	12	0.00	Basic	6 W
Firmicutes	Clostridia	Clostridiales		Flavonifractor	plautii	0.002	0.001	101	12	0.00	Full	6 W
Firmicutes	Erysipelotrichia	Erysipelotrichales	Erysipelotrichaceae	Clostridium	ramosum	0.004	0.002	103	89	0.03	Basic	1 Y
Firmicutes	Erysipelotrichia	Erysipelotrichales	Erysipelotrichaceae	Clostridium	ramosum	0.004	0.002	103	89	0.03	Full	1 Y
Firmicutes	Erysipelotrichia	Erysipelotrichales	Erysipelotrichaceae	Eubacterium	dolichum	0.000	0.000	103	12	0.00	Basic	1 Y
Firmicutes	Erysipelotrichia	Erysipelotrichales	Erysipelotrichaceae	Eubacterium	dolichum	0.000	0.000	103	12	0.01	Full	1 Y
Firmicutes	Negativicutes	Selenomonadales	Veillonellaceae	Veillonella	unclassified	-0.006	0.002	103	87	0.03	Basic	1 Y

[a] MaAsLin models the microbiome as the outcome. Thus, estimate is increase in % relative abundance per point increase on SRS2 T-score
[b] SE: standard error
[c] Number of subjects in analysis
[d] Number of subjects with taxon present and sequenced
[e] Covariates in basic model: age at SRS-2, maternal education, marital status, maternal age, paternal age, child sex; covariates in full model: basic + maternal self-reported smoking during pregnancy, early exclusive breastfeeding, delivery mode, peripartum antibiotics, and gestational age
[f] MB Age: age of microbiome sample; 6W: six weeks; 1Y: one year

Supplementary Table S15. Functional pathways associated with Social Responsiveness Scale-2 scores ($p<0.05$)

Pathway	Estimate[a]	SE[b]	N[c]	N not 0[d]	pval	MB Age[e]	Model[f]	Pathway RA[g]
1CMET2-PWY: N10-formyl-tetrahydrofolate biosynthesis	-1.9E-05	8.35E-06	102	102	0.027	1 Y	Basic	0.03
ALLANTOINDEG-PWY: superpathway of allantoin degradation in yeast	-5E-07	2.2E-07	102	42	0.025	1 Y	Basic	0.000
ANAEROFRUCAT-PWY: homolactic fermentation	-2.5E-05	9.48E-06	102	102	0.009	1 Y	Basic	0.02
ANAEROFRUCAT-PWY: homolactic fermentation	-3.1E-05	9.92E-06	102	102	0.002	1 Y	Full	0.02
ANAGLYCOLYSIS-PWY: glycolysis III (from glucose)	-2.4E-05	1.09E-05	102	102	0.026	1 Y	Basic	0.04
ARGININE-SYN4-PWY: L-ornithine de novo biosynthesis	-2.2E-05	7.22E-06	102	92	0.003	1 Y	Basic	0.002
ARGININE-SYN4-PWY: L-ornithine de novo biosynthesis	-2E-05	7.72E-06	102	92	0.011	1 Y	Full	0.002
ARGININE-SYN4-PWY: L-ornithine de novo biosynthesis	-2.4E-05	9.01E-06	101	92	0.007	6 W	Basic	0.005
ARGININE-SYN4-PWY: L-ornithine de novo biosynthesis	-2.4E-05	9.47E-06	101	92	0.012	6 W	Full	0.005
ASPASN-PWY: superpathway of L-aspartate and L-asparagine biosynthesis	-2.6E-05	7.52E-06	102	102	0.000	1 Y	Basic	0.01
ASPASN-PWY: superpathway of L-aspartate and L-asparagine biosynthesis	-2.5E-05	7.86E-06	102	102	0.001	1 Y	Full	0.01
COLANSYN-PWY: colanic acid building blocks biosynthesis	-8E-06	3.09E-06	102	98	0.011	1 Y	Full	0.003
DAPLYSINESYN-PWY: L-lysine biosynthesis I	-2.5E-05	9.19E-06	102	101	0.008	1 Y	Basic	0.01
DAPLYSINESYN-PWY: L-lysine biosynthesis I	-2.3E-05	9.76E-06	102	101	0.021	1 Y	Full	0.01
FERMENTATION-PWY: mixed acid fermentation	1.31E-05	6.45E-06	101	93	0.045	6 W	Basic	0.005
FERMENTATION-PWY: mixed acid fermentation	1.42E-05	6.89E-06	101	93	0.041	6 W	Full	0.005
GLYCOLYSIS: glycolysis I (from glucose 6-phosphate)	-2.4E-05	1.06E-05	102	102	0.026	1 Y	Full	0.01
GLYCOLYSIS-E-D: superpathway of glycolysis and Entner-Doudoroff	-7.5E-06	3.55E-06	102	100	0.036	1 Y	Full	0.003
HEME-BIOSYNTHESIS-II: heme biosynthesis I (aerobic)	-6E-06	2.34E-06	102	101	0.012	1 Y	Full	0.002
HEMESYN2-PWY: heme biosynthesis II (anaerobic)	-9.3E-06	3.71E-06	102	101	0.014	1 Y	Basic	0.004
HEMESYN2-PWY: heme biosynthesis II (anaerobic)	-9.6E-06	3.99E-06	102	101	0.017	1 Y	Full	0.004
HISDEG-PWY: L-histidine degradation I	1.34E-05	4.99E-06	102	101	0.008	1 Y	Basic	0.002
HISDEG-PWY: L-histidine degradation I	1.17E-05	5.27E-06	102	101	0.028	1 Y	Full	0.002
OANTIGEN-PWY: O-antigen building blocks biosynthesis (E. coli)	-3.1E-05	9.15E-06	102	102	0.000	1 Y	Basic	0.02
OANTIGEN-PWY: O-antigen building blocks biosynthesis (E. coli)	-3.2E-05	9.89E-06	102	102	0.001	1 Y	Full	0.02
P108-PWY: pyruvate fermentation to propanoate I	2.89E-06	1.32E-06	102	67	0.031	1 Y	Basic	0.000
P42-PWY: incomplete reductive TCA cycle	-7.5E-06	3.14E-06	102	102	0.019	1 Y	Basic	0.003
P42-PWY: incomplete reductive TCA cycle	-8.1E-06	3.29E-06	102	102	0.015	1 Y	Full	0.003
P4-PWY: superpathway of L-lysine, L-threonine and L-methionine biosynthesis I	-1.6E-05	6.17E-06	102	101	0.010	1 Y	Basic	0.01
P4-PWY: superpathway of L-lysine, L-threonine and L-methionine biosynthesis I	-1.5E-05	6.55E-06	102	101	0.024	1 Y	Full	0.01
PENTOSE-P-PWY: pentose phosphate pathway	-2.4E-05	7.26E-06	102	102	0.001	1 Y	Basic	0.008
PENTOSE-P-PWY: pentose phosphate pathway	-2.6E-05	7.71E-06	102	102	0.000	1 Y	Full	0.008
PHOSLIPSYN-PWY: superpathway of phospholipid biosynthesis I (bacteria)	-1.7E-05	8.03E-06	102	102	0.038	1 Y	Basic	0.009
PHOSLIPSYN-PWY: superpathway of phospholipid biosynthesis I (bacteria)	-2.4E-05	8.27E-06	102	102	0.004	1 Y	Full	0.009
PRPP-PWY: superpathway of histidine, purine, and pyrimidine biosynthesis	-1.2E-05	5.63E-06	102	101	0.031	1 Y	Basic	0.01
PRPP-PWY: superpathway of histidine, purine, and pyrimidine biosynthesis	-1.5E-05	5.85E-06	102	101	0.013	1 Y	Full	0.01
PWY0-321: phenylacetate degradation I (aerobic)	3.41E-06	1.3E-06	101	18	0.010	6 W	Basic	0.000
PWY0-321: phenylacetate degradation I (aerobic)	3.25E-06	1.37E-06	101	18	0.020	6 W	Full	0.000
PWY0-781: aspartate superpathway	-1.6E-05	6E-06	102	99	0.008	1 Y	Basic	0.009

Pathway	Estimate[a]	SE[b]	N[c]	N not 0[d]	pval	MB Age[e]	Model[f]	Pathway RA[g]
PWY0-781: aspartate superpathway	-1.4E-05	6.4E-06	102	99	0.026	1 Y	Full	0.009
PWY0-845: superpathway of pyridoxal 5'-phosphate biosynthesis and salvage	-2.1E-05	8.83E-06	102	97	0.022	1 Y	Basic	0.01
PWY0-845: superpathway of pyridoxal 5'-phosphate biosynthesis and salvage	-2.6E-05	1.09E-05	101	85	0.020	6 W	Basic	0.008
PWY0-845: superpathway of pyridoxal 5'-phosphate biosynthesis and salvage	-2.6E-05	1.17E-05	101	85	0.030	6 W	Full	0.008
PWY-3001: superpathway of L-isoleucine biosynthesis I	-3.1E-05	1.19E-05	102	102	0.011	1 Y	Basic	0.04
PWY-3001: superpathway of L-isoleucine biosynthesis I	-2.9E-05	1.27E-05	102	102	0.022	1 Y	Full	0.04
PWY-3841: folate transformations II	-5.7E-05	2.31E-05	101	101	0.015	6 W	Basic	0.03
PWY-3841: folate transformations II	-5.4E-05	2.47E-05	101	101	0.032	6 W	Full	0.03
PWY-4242: pantothenate and coenzyme A biosynthesis III	-1.3E-05	6.62E-06	102	102	0.048	1 Y	Basic	0.009
PWY4FS-7: phosphatidylglycerol biosynthesis I (plastidic)	-1.3E-05	6.36E-06	102	102	0.045	1 Y	Basic	0.007
PWY4FS-7: phosphatidylglycerol biosynthesis I (plastidic)	-1.8E-05	6.58E-06	102	102	0.006	1 Y	Full	0.007
PWY4FS-8: phosphatidylglycerol biosynthesis II (non-plastidic)	-1.3E-05	6.36E-06	102	102	0.045	1 Y	Basic	0.007
PWY4FS-8: phosphatidylglycerol biosynthesis II (non-plastidic)	-1.8E-05	6.58E-06	102	102	0.006	1 Y	Full	0.007
PWY-5154: L-arginine biosynthesis III (via N-acetyl-L-citrulline)	-1.9E-05	7.13E-06	102	102	0.010	1 Y	Basic	0.01
PWY-5188: tetrapyrrole biosynthesis I (from glutamate)	2.13E-05	1.07E-05	101	101	0.048	6 W	Basic	0.01
PWY-5188: tetrapyrrole biosynthesis I (from glutamate)	2.4E-05	1.1E-05	101	101	0.032	6 W	Full	0.01
PWY-5367: petroselinate biosynthesis	1.28E-05	5.99E-06	102	93	0.035	1 Y	Basic	0.004
PWY-5484: glycolysis II (from fructose 6-phosphate)	-2.2E-05	9.9E-06	102	102	0.028	1 Y	Full	0.01
PWY-5676: acetyl-CoA fermentation to butanoate II	-4.7E-06	2.23E-06	102	100	0.039	1 Y	Full	0.002
PWY-5695: urate biosynthesis/inosine 5'-phosphate degradation	-2.4E-05	1.15E-05	102	102	0.038	1 Y	Basic	0.03
PWY-5791: 1,4-dihydroxy-2-naphthoate biosynthesis II (plants)	-7.6E-06	3.13E-06	102	99	0.017	1 Y	Basic	0.002
PWY-5791: 1,4-dihydroxy-2-naphthoate biosynthesis II (plants)	-7.6E-06	3.31E-06	102	99	0.023	1 Y	Full	0.002
PWY-5837: 1,4-dihydroxy-2-naphthoate biosynthesis I	-7.6E-06	3.13E-06	102	99	0.017	1 Y	Basic	0.002
PWY-5837: 1,4-dihydroxy-2-naphthoate biosynthesis I	-7.6E-06	3.31E-06	102	99	0.023	1 Y	Full	0.002
PWY-5897: superpathway of menaquinol-11 biosynthesis	-1.3E-05	4.46E-06	102	99	0.004	1 Y	Basic	0.004
PWY-5897: superpathway of menaquinol-11 biosynthesis	-1.3E-05	4.71E-06	102	99	0.007	1 Y	Full	0.004
PWY-5898: superpathway of menaquinol-12 biosynthesis	-1.3E-05	4.46E-06	102	99	0.004	1 Y	Basic	0.004
PWY-5898: superpathway of menaquinol-12 biosynthesis	-1.3E-05	4.71E-06	102	99	0.007	1 Y	Full	0.004
PWY-5899: superpathway of menaquinol-13 biosynthesis	-1.3E-05	4.46E-06	102	99	0.004	1 Y	Basic	0.004
PWY-5899: superpathway of menaquinol-13 biosynthesis	-1.3E-05	4.71E-06	102	99	0.007	1 Y	Full	0.004
PWY-6147: 6-hydroxymethyl-dihydropterin diphosphate biosynthesis I	-1.6E-05	6.99E-06	102	102	0.028	1 Y	Full	0.006
PWY-6897: thiamin salvage II	-2.5E-05	7.84E-06	102	102	0.001	1 Y	Basic	0.01
PWY-6897: thiamin salvage II	-2.2E-05	8.31E-06	102	102	0.009	1 Y	Full	0.01
PWY-6901: superpathway of glucose and xylose degradation	-1.1E-05	5E-06	102	102	0.036	1 Y	Basic	0.005
PWY-6901: superpathway of glucose and xylose degradation	-1.4E-05	5.25E-06	102	102	0.009	1 Y	Full	0.005
PWY-7204: pyridoxal 5'-phosphate salvage II (plants)	-1.7E-05	6.44E-06	101	72	0.011	6 W	Basic	0.004
PWY-7204: pyridoxal 5'-phosphate salvage II (plants)	-1.6E-05	6.76E-06	101	72	0.023	6 W	Full	0.004
PWY-7282: 4-amino-2-methyl-5-phosphomethylpyrimidine biosynthesis (yeast)	-2.6E-05	9.4E-06	102	99	0.006	1 Y	Basic	0.01
PWY-7282: 4-amino-2-methyl-5-phosphomethylpyrimidine biosynthesis (yeast)	-2.3E-05	1.01E-05	102	99	0.026	1 Y	Full	0.01
PWY-7323: superpathway of GDP-mannose-derived O-antigen building blocks biosynthesis	-8.2E-06	3.82E-06	102	97	0.034	1 Y	Full	0.003

Pathway	Estimate[a]	SE[b]	N[c]	N not 0[d]	pval	MB Age[e]	Model[f]	Pathway RA[g]
PWY-7328: superpathway of UDP-glucose-derived O-antigen building blocks biosynthesis	5.67E-06	2.81E-06	101	65	0.047	6 W	Full	0.002
PWY-7323: superpathway of GDP-mannose-derived O-antigen building blocks biosynthesis	-8.2E-06	3.82E-06	102	97	0.034	1 Y	Full	0.003
PWY-7328: superpathway of UDP-glucose-derived O-antigen building blocks biosynthesis	5.67E-06	2.81E-06	101	65	0.047	6 W	Full	0.002
PWY-7357: thiamin formation from pyrithiamine and oxythiamine (yeast)	-2.3E-05	1.03E-05	102	102	0.027	1 Y	Basic	0.02
PWY-7383: anaerobic energy metabolism (invertebrates, cytosol)	9.36E-06	4.2E-06	101	79	0.028	6 W	Basic	0.002
PWY-7663: gondoate biosynthesis (anaerobic)	-2.5E-05	7.98E-06	102	102	0.002	1 Y	Basic	0.02
PWY-7663: gondoate biosynthesis (anaerobic)	-2.4E-05	8.63E-06	102	102	0.006	1 Y	Full	0.02
PYRIDOXSYN-PWY: pyridoxal 5'-phosphate biosynthesis I	-1.4E-05	7.09E-06	102	99	0.047	1 Y	Basic	0.008
PYRIDOXSYN-PWY: pyridoxal 5'-phosphate biosynthesis I	-1.9E-05	8.86E-06	101	96	0.038	6 W	Basic	0.008
THRESYN-PWY: superpathway of L-threonine biosynthesis	-3.6E-05	1.28E-05	102	102	0.006	1 Y	Basic	0.03
THRESYN-PWY: superpathway of L-threonine biosynthesis	-3.4E-05	1.37E-05	102	102	0.015	1 Y	Full	0.03

[a] MaAsLin models the microbiome as the outcome. Thus, estimate is increase in % relative abundance per point increase on SRS2 T-score
[b] SE: standard error
[c] Number of subjects in analysis
[d] Number of subjects with pathway present
[e] MB Age: age of microbiome sample; 6W: six weeks; 1Y: one year
[f] Covariates in basic model: age at SRS-2, maternal education, marital status, maternal age, paternal age, child sex; covariates in full model: basic + maternal self-reported smoking during pregnancy, early exclusive breastfeeding, delivery mode, peripartum antibiotics, and gestational age
[g] Pathway RA: pathway relative abundance

Supplementary Table S16. Sample sizes and model variables for primary and sensitivity analyses

Microbiome Age	Primary[a]	Term Infants[a]	Cotinine[b]	Any Breast Feeding at Six Months[c]	PRQ[d]	Metagenomics
Six Weeks	166	153	149	163	166	101
One Year	158	145	145	156	156	103
Two Years	129	120	118	127	127	–
Three Years	140	129	129	136	138	–
All	21	–	–	–	–	–

[a] Sample sizes are the same for basic and full models. Covariates in basic model: age at SRS-2, maternal education, marital status, maternal age, paternal age, child sex; covariates in full model: basic + maternal self-reported smoking during pregnancy, early exclusive breastfeeding, delivery mode, peripartum antibiotics, and gestational age

[b] Sample sizes are the same for basic and full models. Covariates the same as primary model except full model adjusts for maternal urinary cotinine during pregnancy in place of maternal self-report of smoking

[c] Sample sizes are the same for basic and full models. Covariates the same as primary model with the addition of an indicator variable for any breastfeeding at six months in the basic and full models for six-week microbiome analyses and in the full model for all other microbiome age analyses

[d] Sample sizes are the same for basic and full models. Covariates the same as primary model with the addition of the Parenting Relationship Questionnaire (PRQ)

Supplementary Figure S1. Volcano plot of associations between metagenomically identified functional pathways and SRS2 total T-scores

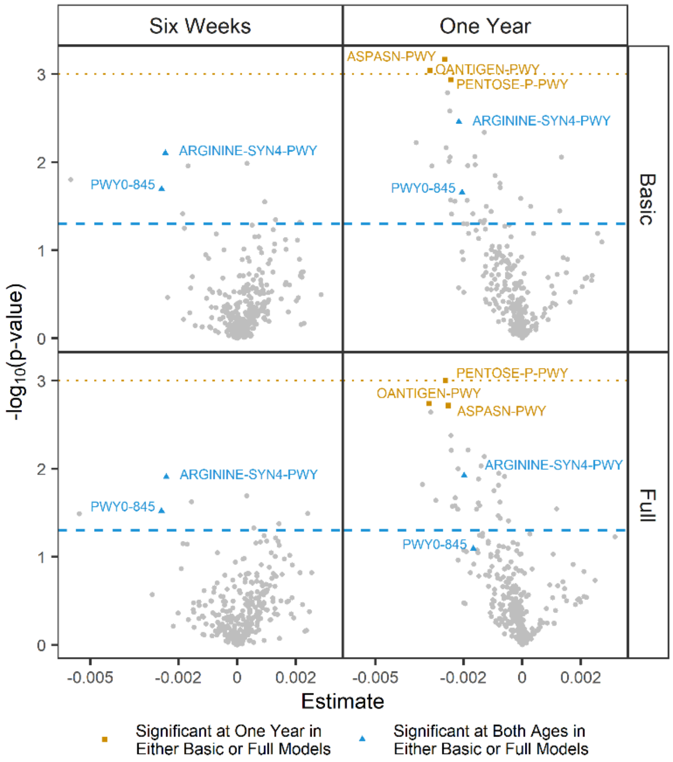

Points representing unmapped or unintegrated pathways have been removed for visual clarity (2 points per plot). Basic models adjust for age at SRS2, maternal education, marital status, maternal age, paternal age, and child sex. Full models adjust for Basic Models + maternal self-reported smoking during pregnancy, early exclusive breastfeeding, delivery mode, peripartum antibiotics, and gestational age. Dashed blue line indicates p = 0.05, dotted orange line indicates p = 0.001. ARGININE-SYN4-PWY: L-ornithine de novo biosynthesis; ASPASN-PWY: superpathway of L-aspartate and L-asparagine synthesis; OANTINGEN-PWY: O-antigen building blocks biosynthesis (*Escherichia coli*); PENTOSE-P-PWY: pentose phosphate pathway; PWY0-845: superpathway of pyridoxal 5’-phosphate biosynthesis and salvage.

Supplementary Figure S2. Selection of subjects into analytical cohort

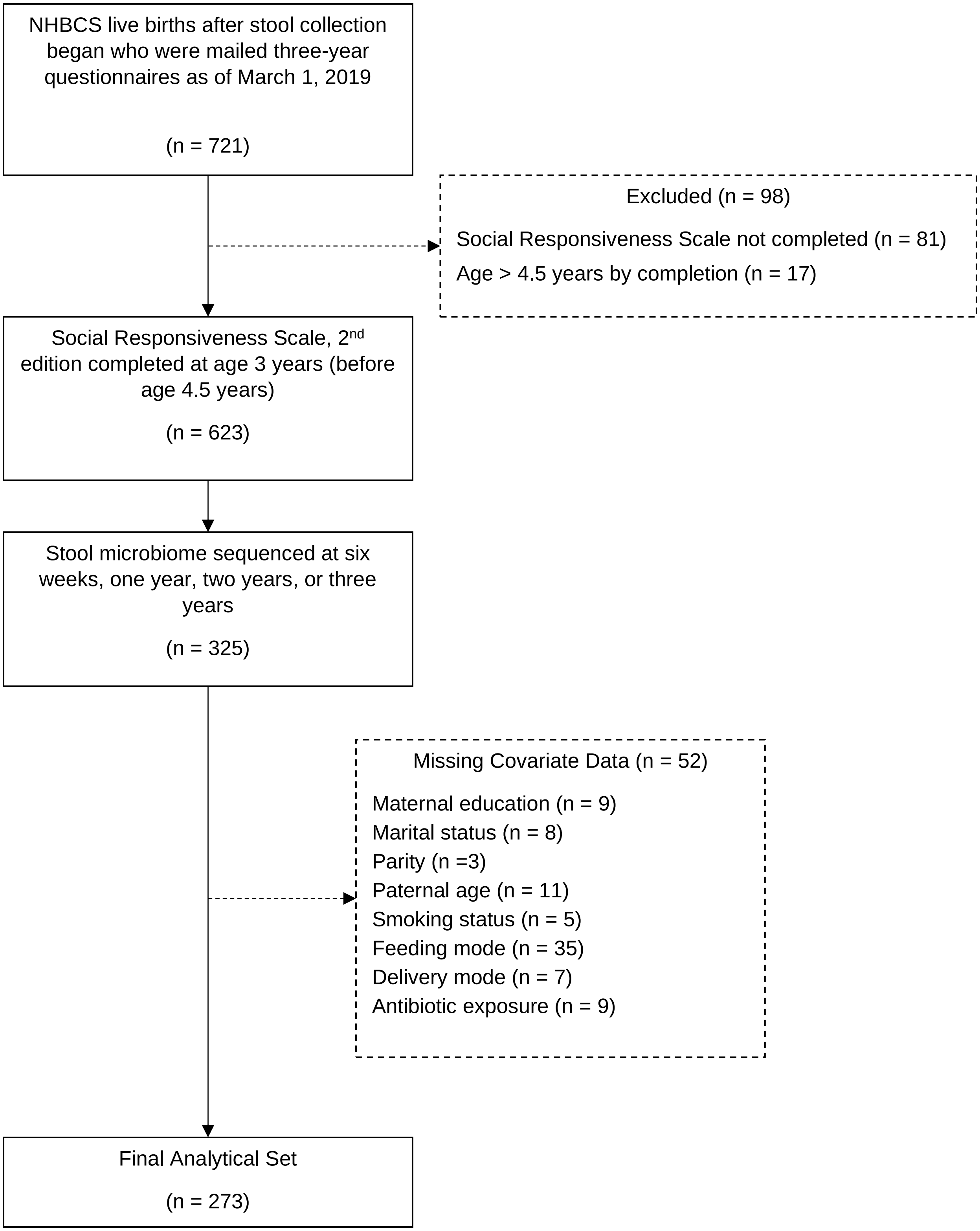

Supplement: Supplementary file 1 — Supplementary information. [file 41598_2020_72386_MOESM1_ESM.pdf]
